# From Biological Mechanisms to Task-Oriented Design Principles: A Critical Review of Fish-like Biomimetic Robots

**DOI:** 10.3390/biomimetics11090630

**Published:** 2026-09-03

**Authors:** Bo Yan, Hongyuan Liu, Decai Tang

**Affiliations:** 1 Department of Shipping Engineering, Sichuan Vocational and Technical College of Communications, Chengdu 611130, China; yanbo@svtcc.edu.cn; 2 School of Engineering, Westlake University, Hangzhou 310030, China

**Keywords:** robotic fish, biomimetic locomotion, bioinspired propulsion, compliance, variable stiffness, embodied autonomy, task-oriented evaluation

## Abstract

Fish-like biomimetic robots increasingly combine compliant structures, soft and variable-stiffness actuation, distributed sensing, and autonomous control, but cross-study comparison remains difficult because biological inspiration, robotic embodiment, test boundaries, and mission definitions are heterogeneous. We synthesize the field through a mechanism-to-evidence framework that links biological mechanisms to measurable descriptors, robotic embodiment, controlled interventions, task-oriented evidence, and conditional design principles. Across the literature, the transferable unit is not external resemblance but a functional mechanism whose advantage remains measurable after robotic integration. Three conclusions recur: dynamic performance depends on matching stiffness, actuation frequency, damping, and fluid loading within the intended operating range; morphology, sensing, control, power, and payload must be co-designed; and component, free-swimming, controlled-task, and field studies support different scopes of inference. We translate these findings into nine evidence-informed conditional design principles with explicit applicability limits and discriminating validation tests. Major gaps remain in wet-state dynamic characterization, complete reporting of power boundaries and kinematics, uncertainty and failures, matched task-level comparisons, and long-duration field validation. The resulting framework shifts evaluation from peak metrics and taxonomic labels toward task-conditioned, evidence-bounded design decisions.

## 1. Introduction

### 1.1. Why Fish-like Robots Require Evidence-Informed Conditional Design Principles

Fish-like biomimetic robots sit at the intersection of comparative biology, fluid mechanics, soft materials, and underwater engineering. Their engineering value, however, does not follow from resemblance alone. Fish swimming emerges from the coupled interaction of body geometry, muscle activation, passive elasticity, fin motion, fluid loading, and sensory feedback. A robot can therefore appear fish-like while embodying little of the functional mechanism that underpins biological locomotion. Conversely, a mechanically simplified swimmer can reproduce a useful principle—such as posterior-body wave amplification, passive fin feathering, or pressure-gradient sensing—without closely imitating a particular species. This distinction has become more important as robotic fish have expanded from rigid serial-link prototypes to compliant, soft, variable-stiffness, sensor-integrated, and reconfigurable systems [1,2].

Fish-like propulsion is not a universal replacement for conventional autonomous underwater vehicles or remotely operated vehicles. Propeller-driven systems remain preferable for many missions that prioritize long-range transit, large payloads, mature navigation, or high-thrust station keeping. Fish-like propulsion becomes compelling when the mission instead values low-speed maneuverability, reduced mechanical hazard, continuous body deformation, biologically compatible motion, or operation near complex boundaries. Representative settings include close-range ecological observation, inspection of aquaculture facilities and submerged structures, environmental sensing and sampling, animal-behavior experiments, and distributed sensing by multiple robots. The central engineering question is therefore whether a specified biological mechanism produces a measurable advantage for a defined task, scale, and experimental boundary.

Existing reviews have organized the field by swimming mode, animal archetype, actuator, body architecture, sensing modality, controller, or application [3,4]. These taxonomies remain valuable, but they do not by themselves explain when a biological mechanism becomes transferable design knowledge. Two platforms labeled carangiform may differ in active body length, joint distribution, fin stiffness, amplitude envelope, and power boundary, whereas systems inspired by different animals may exploit the same engineering mechanism, such as passive elastic recoil or a posterior stiffness gradient. A design-oriented synthesis must therefore connect biological function to measurable descriptors, show how those descriptors are embodied, identify the evidence context in which the mechanism is tested, and determine whether the effect persists at the level of robot capability or task outcome.

This review uses that mechanism-to-evidence relation as its central unit of synthesis. The chain links a biological mechanism to a measurable descriptor, robotic embodiment, controlled intervention, task-oriented evidence, and an evidence-informed conditional design principle. This organization complements prior reviews of BCF locomotion, implementation, and autonomy rather than repeating their taxonomies [5,6,7].

Table 1 positions the present critical review relative to representative prior reviews. The comparison clarifies differences in organizing emphasis; it is not intended as an exhaustive bibliometric ranking. Earlier reviews provide indispensable taxonomies and broad technical coverage. The added focus here is the explicit connection among a proposed mechanism, an intervention or comparison, its evidence context, a task-relevant endpoint, and the conditions under which the resulting design principle may fail.

#### 1.1.1. From Mechanical Imitation to Functional Transfer

Early robotic fish established that serial joints, oscillating tails, and periodic control laws could generate repeatable fish-like motion and provide controllable platforms for gait and hydrodynamic studies [9,10]. Subsequent work showed that commanded joint trajectories describe only part of the realized swimming state. Flexible structures deform under water loading; fins alter camber and angle of attack passively; transmissions introduce compliance, backlash, and delay; and payload, buoyancy, and sealing modify the inertia and stability of the complete robot. The same nominal gait can consequently produce different body waves, force histories, and energy use on different platforms. Integrative mechanical designs and fin-propelled vehicles further demonstrate that locomotion emerges from the interaction of mechanism, structure, and control [11,12].

A function-oriented approach, therefore, begins with the capability to be transferred. Fast transit may depend on active body length, posterior stiffness, peduncle geometry, tail-beat frequency, and fin recoil. Low-speed inspection may instead be limited by paired-fin stroke, body stability, field of view, sensor placement, and station-keeping control. Biological interaction adds wake disturbance, motion regularity, approach direction, acoustic output, and contact safety. A visually faithful outer shape can still be functionally poor if the payload housing suppresses required deformation, shifts inertia, or introduces protruding interfaces. Because same-platform experiments that isolate visual fidelity as the sole intervention remain scarce, this review treats visual resemblance as a design variable rather than assuming either a universal benefit or penalty.

Robotic fish also serve as controllable physical models of biological locomotion [13]. Biological swimmers are difficult to perturb one variable at a time because morphology, muscle activation, sensory feedback, and behavior change together. Robotic platforms can vary stiffness, fin geometry, phase, or sensor placement while holding much of the remaining system approximately constant. Such within-platform perturbations support stronger causal interpretation than comparisons among unrelated robots. Tuna-inspired experimental platforms, flexible-body variants, and reconfigurable systems illustrate this physical-model role [14,15,16].

#### 1.1.2. Evidence Contexts and Unavoidable Trade-Offs

The literature spans theoretical models, actuator coupons, isolated fins, tethered swimmers, free-swimming tank tests, controlled tasks, biological-interaction studies, and field deployments. These experiments answer different questions. A material test can establish strain or bandwidth; a fin rig can establish thrust; a free-swimming robot can establish integrated locomotion; and a field experiment can establish feasibility under realistic operating constraints. Their value is complementary, but their scopes of inference differ. Component deformation, maximum speed, and a successful field mission therefore support different claims about mechanism, capability, and readiness.

The same distinction applies to design trade-offs. A high-frequency, tail-dominant architecture may favor rapid cruising but demand precise dynamic matching. A fully soft body may reduce contact forces and generate smooth curvature while constraining payload, force density, or state estimation. More joints enlarge the controllable gait space but add sealing, wiring, calibration, and failure points. Flow sensing can improve environmental awareness while increasing self-generated-flow contamination and waterproofing requirements. The relevant design target is thus a task-conditioned compromise, not a globally optimal robotic fish.

Four broad mission emphases recur across the literature. *Transit-oriented* systems prioritize normalized speed, energy use, and directional stability. *Interaction-oriented* systems prioritize mechanical safety, low disturbance, and biological response. *Inspection, monitoring, and sampling* systems prioritize positioning, payload, sensor quality, and reliable low-speed operation. *Collective systems* add communication, distributed perception, scalability, and failure tolerance. These categories overlap, but they expose different bottlenecks and provide a more informative basis for evaluation than a single ranking by peak speed.

### 1.2. Scope, Objectives, and Contributions

This review focuses on robotic fish and fish-like underwater robots whose propulsion or maneuvering is generated primarily through body, caudal-fin, median-fin, or paired-fin motion. It covers rigid multi-joint architectures, rigid–soft hybrids, fully soft platforms, passive and variable compliance, motorized and smart-material actuation, artificial lateral-line sensing, model-based and learning-enabled control, robot–fish interaction, and task-oriented deployment. Biological and hydrodynamic studies are included when they define mechanisms or variables that can be tested with robotic platforms. Non-fish aquatic robots are considered only when they provide a clearly transferable principle; the article is not intended as a general survey of soft underwater robotics.

Five questions guide the synthesis:

1. Which fish locomotion and sensing mechanisms can be represented as explicit engineering descriptors?
2. How do morphology, actuation, compliance, sensing, control, power, and payload interact in the realized robot?
3. What evidence is required before a component effect can be interpreted as a robot-level or mission-level advantage?
4. Which metrics are appropriate for different robotic fish tasks, and why is a single speed ranking inadequate?
5. Which recurring findings can be reformulated as evidence-informed conditional design principles?

The review addresses these questions through four linked contributions. It first develops a mechanism-based framework that connects biological observations with measurable engineering variables and evidence contexts. It then reorganizes platform, actuation, sensing, control, and application evidence around measurable outcomes rather than broad qualitative rankings. A layered task-oriented evaluation framework separates mechanism-level descriptors, robot-level capabilities, and task-level outcomes. Finally, recurring observations are reformulated as conditional design principles with explicit applicability limits, counterevidence, and discriminating validation tests. The aim is not to prescribe a single architecture but to make the assumptions, evidence, and failure conditions behind design choices explicit.

### 1.3. Mechanism-Based Structural Design Framework

**Figure 1:**
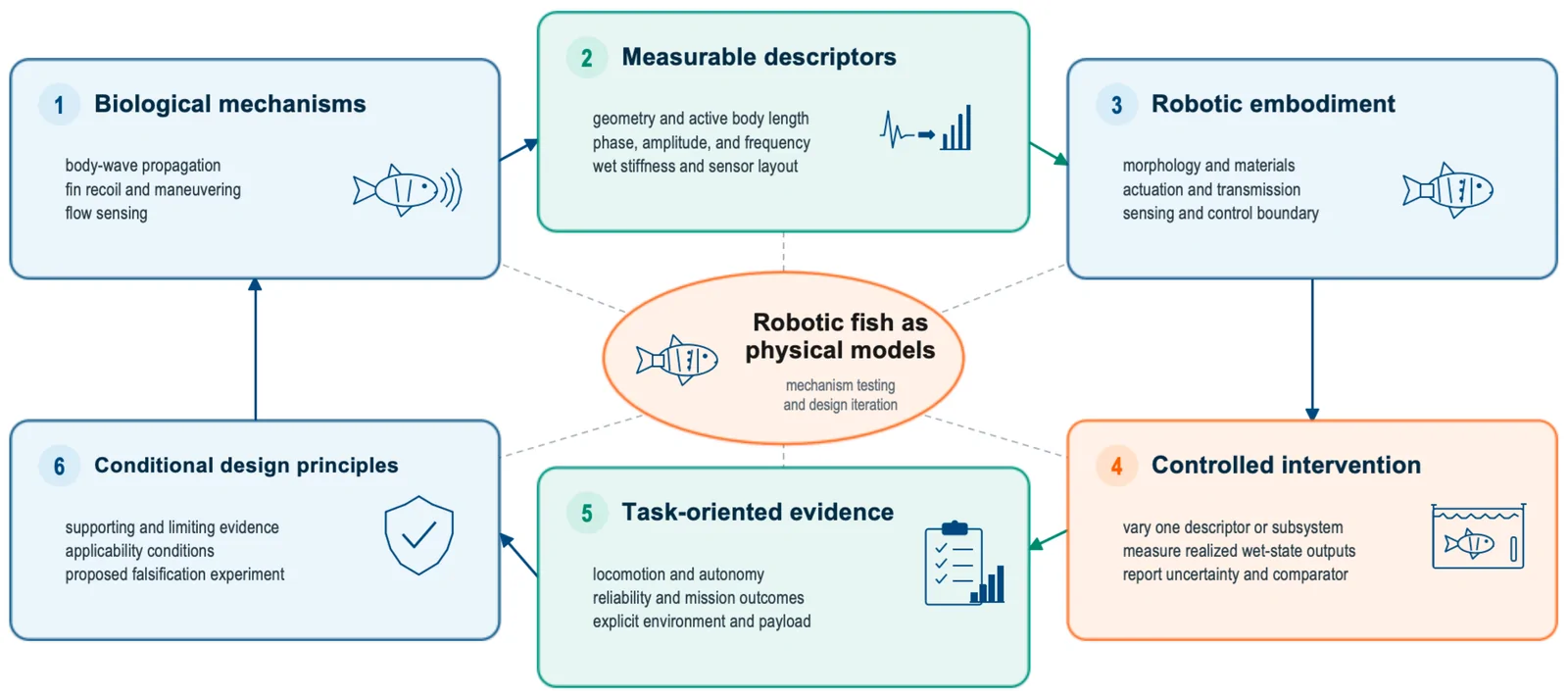
Mechanism-based structural design framework for fish-like biomimetic robots. Functional mechanism transfer progresses from biological mechanism, through measurable descriptors and robotic embodiment, to controlled intervention, task-oriented evidence, and a bounded conditional design principle. Biological locomotion and sensing functions are abstracted into measurable descriptors and embodied through morphology, actuation, materials, sensing, and control. Controlled experiments connect implementation to task-oriented evidence, while the results feed back into refined evidence-informed conditional design principles. Robotic fish play a dual role as engineered underwater systems and controllable physical models for mechanism testing.

Figure 1 summarizes the six-stage framework used throughout the review. Functional mechanism transfer progresses from a biological mechanism to measurable descriptors, robotic embodiment, controlled intervention, task-oriented evidence, and a bounded conditional design principle. External resemblance alone does not complete this chain; the mechanism must remain observable after embodiment and must be tested against an appropriate comparator or task boundary.

The framework is iterative rather than strictly linear. A compliant-tail experiment may simultaneously inform structural implementation, hydrodynamic mechanism, and robot-level performance. A pressure-sensor array may establish a sensing principle before its task-level value is demonstrated. A field robot may establish system integration while leaving the contribution of an individual component unresolved. Making these distinctions explicit allows promising results to be interpreted without extrapolating beyond the available evidence.

#### 1.3.1. Evidence Context and Table-Level Provenance

The framework uses five non-ordinal evidence-context labels to align each claim with the experiment that supports it: EC-M (model/theory), EC-C (component or tethered intervention), EC-R (integrated free-swimming robot), EC-T (controlled task), and EC-F (field or biological interaction). These labels describe inference scope, not study quality or degree of onboard autonomy. Table 2 summarizes the associated claim boundaries and the reporting features that strengthen transferability.

#### 1.3.2. Notation

Table 3 defines the structural, hydrodynamic, and energetic symbols used throughout the article. The bending-rigidity notation is standardized as EI(x), and the wet damping ratio is consistently denoted by $\zeta_{w}$.

For numerical values in the intervention evidence tables, R denotes a directly reported value, D a transparent derivation from reported quantities, G a graph-digitized value, NR a value that is not reported or not unambiguously traceable, and IC a value that should not be compared numerically because the protocol or definition is incompatible. These labels describe table-level provenance only; they are not combined into a review-wide score. Cross-platform normalized values are interpreted descriptively unless definitions and test boundaries match, whereas matched within-platform interventions are given greater causal weight.

The remainder of the article follows this logic. Section 2 translates swimming modes and hydrodynamics into engineering descriptors. Section 3 examines their embodiment through morphology, actuation, and compliance. Section 4 analyzes sensing, control, and autonomy. Section 5 connects mechanism descriptors to robot capabilities and task outcomes. Section 6 consolidates conditional design principles, unresolved contradictions, and the experiments most likely to resolve them.

## 2. Biological Mechanisms and Engineering Descriptors

### 2.1. Swimming Modes as Distributions of Deformation

Fish locomotion is commonly divided into body-and/or-caudal-fin (BCF) and median-and/or-paired-fin (MPF) modes. The distinction remains useful, but for engineering it is more informative to interpret each mode as a spatial distribution of deformation and control authority. Anguilliform swimming distributes bending over most of a slender body; subcarangiform and carangiform swimming progressively concentrate deformation toward the posterior region; thunniform swimming combines a comparatively stable anterior body with concentrated caudal action; and ostraciiform swimming relies primarily on tail oscillation from an otherwise rigid body [17]. This descriptor-based interpretation avoids treating the biological label as a sufficient specification.

These categories suggest different robotic architectures. Distributed undulation supports obstacle negotiation and large body curvature, but usually increases actuator count, sealing points, and control dimensionality. Posterior-dominant BCF motion reduces the number of actively controlled segments and provides a practical compromise between speed and maneuverability. A thunniform architecture can access high-frequency and high-speed operation, but it requires careful matching of body stiffness, tail kinematics, and actuator bandwidth. An ostraciiform robot offers mechanical simplicity, although its reduced gait space limits maneuvering and flow adaptation. MPF mechanisms provide complementary control for hovering, turning, braking, and posture regulation; they are therefore particularly relevant to inspection and biological interaction tasks.

The categories are not mutually exclusive design choices. A task-oriented robot may use BCF propulsion for transit and pectoral or median fins for local maneuvering. Mechanism-design methods for BCF locomotion further illustrate how a biological category can be converted into explicit kinematic and structural parameters rather than treated as a visual label [18]. A variable-stiffness platform may reproduce several nominal swimming modes without changing its overall outline. The engineering value of a biological label therefore depends on whether the realized curvature distribution, phase lag, frequency, and fin motion are reported.

#### 2.1.1. Anguilliform and Subcarangiform Distributions

Anguilliform motion distributes large-amplitude bending over much of the body. In engineering terms, this requires either many controlled joints or a continuum that can sustain a traveling curvature wave. The distributed motion can provide strong turning authority and the ability to conform to confined geometry, but it also increases lateral motion of the body and the dimensionality of gait control. A rigid serial mechanism must coordinate phase among many links, whereas a continuum design must manage the dependence of realized curvature on material stiffness, damping, and water loading. The resulting choice is not simply between more and fewer actuators; it is between actively specifying the body wave and allowing part of that wave to emerge from passive dynamics.

Subcarangiform motion concentrates deformation progressively toward the posterior body. This distribution is attractive for general-purpose robotic fish because it leaves a relatively stable anterior compartment for batteries, computation, buoyancy control, and sensors while preserving a compliant propulsive region. It also creates a natural interface for rigid–soft hybrid design. The engineering challenge is to avoid a sharp impedance discontinuity between the payload-bearing head and the flexible tail. If the transition is too stiff, the curvature localizes at one joint; if it is too soft, the tail may lag excessively or dissipate energy. A graded backbone or distributed compliance can provide a smoother transfer of the bending moment.

#### 2.1.2. Carangiform, Thunniform, and Ostraciiform Specializations

Carangiform systems further restrict large-amplitude motion toward the tail, reducing lateral excursion of the anterior body and simplifying sensor stabilization. The reduced body participation can improve straight-line stability and packaging, but performance becomes more sensitive to peduncle and fin design. Thunniform systems represent the most tail-dominant extreme. High performance requires not only a high tail-beat frequency but also a structural response that produces an appropriate trailing-edge amplitude and phase. The Tunabot family demonstrates the value of an experimental platform in which frequency and body flexibility can be varied while kinematics and energetic outcomes are measured [14,15]. Such platforms show why a named gait is insufficient: the effective wave depends on stiffness, actuator dynamics, and fluid load.

Ostraciiform propulsion maintains a comparatively rigid body and oscillates the caudal fin with few degrees of freedom. The resulting architecture can be compact and robust, which is attractive for small robots and educational or sensing platforms. Its limitations are equally clear. A single tail joint provides limited control over body-wave shape, and turning often requires asymmetric tail motion, auxiliary fins, or body reorientation. The MARCO platform remains an important example because it connects hydrodynamic analysis, mechanism design, and fabrication within a low-degree-of-freedom architecture [10]. Segmented caudal-fin designs further show that passive structural tailoring can expand thrust range and resonance behavior without adding an equivalent number of actuators [19].

#### 2.1.3. MPF-Dominant Control and Mixed-Mode Robots

Median- and paired-fin propulsion is especially relevant when the task requires hovering, braking, reversing, depth regulation, or precise approach. Flexible pectoral fins can use passive feathering to align with fluid load and reduce the number of actively controlled variables [20]. Folding and spanwise deformation further expand the achievable force directions [21]. Robotic fin and ray-inspired studies also demonstrate that complex biological morphology can be reduced to a smaller set of stroke, curvature, stiffness, and force-direction variables [12,22]. These mechanisms are often discussed separately from BCF propulsion, yet many practical robots combine them: a caudal or posterior body mode provides transit, while pectoral, dorsal, or anal fins provide trim and local maneuvering. This mixed-mode interpretation is more useful for engineering than forcing a platform into one biological category.

The design consequence is that locomotion mode should be represented by a spatial distribution of deformation and control authority. A concise descriptor set includes the fraction of the body participating in undulation, the amplitude envelope, the phase or wave speed, the location of active and passive joints, and the fins available for force-vector control. These variables can be compared across platforms even when the biological labels differ.

### 2.2. Biological Sensing and Neuromechanical Coupling

Locomotion in living fish is not generated by morphology and muscle in isolation. Visual, vestibular, proprioceptive, and lateral-line information continuously modifies body and fin activation. For engineering, the relevant lesson is not that every biological sensor must be copied, but that propulsion, sensing, and mechanical response form one feedback system. A robot that reproduces body-wave kinematics without considering how deformation and flow are observed captures only the feedforward part of the biological mechanism.

The lateral line is especially relevant because it measures local pressure and water motion close to the body. Biological fish use this distributed information for behaviors that include flow orientation, obstacle response, prey detection, and schooling. Artificial lateral-line research has translated the mechanism into pressure and flow arrays, but the transfer is not purely electronic: sensor location, body geometry, boundary layer, and self-generated motion determine the available signal [23,24]. The biological mechanism is therefore better represented by a sensing distribution and an inference task than by the presence of a pressure sensor alone.

Neuromechanical coupling also affects stiffness. Biological muscle activation changes both force production and body impedance, while tendons, skin, vertebral structures, and fins redistribute load. Robotic work that varies body stiffness demonstrates that mechanical tuning changes the realized wave and the fluid reaction even when the nominal actuation command remains similar [25,26]. This observation motivates variable-stiffness robots, but it also cautions against interpreting a passive elastic optimum as a universal biological analogue. The relevant descriptors are spatial stiffness, damping, activation timing, and their dependence on frequency and external load.

Sensory and mechanical mechanisms can therefore be encoded through three complementary descriptor groups. The first describes what the body can feel: pressure distribution, strain field, inertial state, and visual or acoustic context. The second describes what can be changed: motor phase, fin stroke, body stiffness, and gait mode. The third describes the feedback pathway: latency, estimator, controller, and the body dynamics through which the response is expressed. These groups establish the conceptual bridge from biological inspiration in this section to the embodied autonomy discussed in Section 4.

### 2.3. Hydrodynamic Mechanisms

The central hydrodynamic problem is the conversion of lateral deformation into net momentum in the swimming direction. Elongated-body theory explains how reactive forces associated with accelerated fluid contribute to propulsion in slender swimmers [27]. Oscillating-foil and self-propelled robotic studies further show that frequency, amplitude, phase, fin compliance, and wake organization interact to determine thrust and efficiency [28,29]. These theories provide useful baselines, but a robotic body introduces finite joint spacing, actuator inertia, damping, skin deformation, and control constraints that can alter the realized wave.

A prescribed joint command is therefore not equivalent to the actual body kinematics. In a rigid multi-joint fish, motor trajectories largely determine local angles, although backlash and hydrodynamic load still matter. In a compliant body, actuator input interacts with structural stiffness and water loading to produce the final curvature field. This interaction can improve performance when passive deformation reinforces the desired traveling wave, but excessive compliance can reduce thrust, delay response, or dissipate energy. Robotic and computational studies have demonstrated that body stiffness changes the balance among internal force, fluid reaction, and waveform propagation [25].

Caudal-fin mechanics are similarly coupled. A fin may act as a rigid lifting surface, an anisotropic flexible plate, or a membrane whose effective angle of attack emerges from fluid loading. Passive feathering can reduce control complexity in pectoral fins, while active or passive tail flexion can shift the phase between anterior and posterior motion [20,30]. The relevant descriptors are not simply “flexible” or “rigid” but bending stiffness, torsional stiffness, damping, anisotropy, attachment geometry, and the ratio between forcing frequency and structural natural frequency.

The wake structure is useful as a diagnostic, not as a stand-alone performance score. Separating drag and thrust is itself a modeling choice that requires a stated body and propulsor boundary [31]. Counter-propagating fin waves and undulating-fin experiments illustrate that similar net motions can be produced through distinct local wave interactions and force distributions [32,33]. Alternating vortices and momentum jets can reveal whether the chosen gait produces thrust, but visually similar wakes may occur under different power inputs or body dynamics. Wake measurements are most informative when paired with force, kinematics, and energy accounting. This is one reason why physical robotic models are valuable: a platform can reproduce a gait repeatedly while stiffness, frequency, or fin geometry is varied.

#### 2.3.1. Body-Wave Generation and Reactive Force

In elongated-body descriptions, lateral acceleration of the body and the associated added-mass reaction contribute to axial momentum transfer [27]. A robotic implementation must therefore reproduce more than a static sinusoidal centerline. The local curvature, lateral velocity, and phase progression determine how momentum is exchanged along the body. Errors in phase can generate large lateral force without useful thrust, while excessive anterior motion can increase recoil and disturb onboard sensing. For a discrete robot, the commanded joint phases approximate a continuous wave; for a compliant robot, the commanded input interacts with structural modes to produce the observed wave. Reporting both commanded and measured kinematics is thus essential.

#### 2.3.2. Caudal-Fin Interaction and Wake Organization

The caudal fin converts posterior-body motion into a combination of lift-based and added-mass forces. Frequency, amplitude, pitch, flexibility, and planform determine the resulting force and wake. Oscillating-foil research links efficient thrust production to coordinated kinematics and wake organization, often summarized through the Strouhal number [28]. However, the same Strouhal value can arise from different combinations of frequency, amplitude, and speed, and it does not capture fin stiffness, the angle of attack, or transient behavior. It should therefore be used to describe a regime rather than as a universal efficiency score.

A reverse Kármán-like wake is frequently treated as a visual signature of thrust, but wake appearance alone does not establish whole-robot efficiency. The momentum distribution, three-dimensional vortex topology, body drag, and mechanical or electrical input must also be considered. Wake measurements are most informative when synchronized with body kinematics and force or free-swimming speed. This is one reason controlled robotic physical models are valuable: they can vary stiffness or motor program while preserving geometry and test conditions.

#### 2.3.3. Fluid–Structure Interaction and Resonance

Compliance changes the mapping from actuator command to body and fin motion. Near an appropriate dynamic operating point, elastic deformation can amplify posterior motion, reduce actuator effort, and smooth the body wave. Away from that point, the same compliance can introduce lag, excessive curvature, or energy dissipation. Studies that vary stiffness within a common platform provide stronger evidence than comparisons between unrelated rigid and soft robots. Tunabot Flex, for example, showed that flexibility can improve both speed and transport cost under tested conditions, while computational and robotic analyses emphasize that the effect depends on the relationship among stiffness, frequency, and body mode [15,25].

Active and passive tail flexion provide another route to shaping the interaction. A controlled flexion schedule can cooperate with hydrodynamic loading rather than resist it, but it also introduces sensing and actuation demands [30]. The relevant design variable is therefore not maximum flexibility; it is the phase-dependent mechanical response at the operating point. This response may be characterized through frequency sweeps, modal analysis, measured curvature, or an effective stiffness identified under water.

#### 2.3.4. Maneuvering, Braking, and Unsteady Flow

Steady forward swimming receives disproportionate attention because it is easier to standardize, yet many target tasks are dominated by unsteady maneuvers. Turning requires asymmetric force generation, body curvature, or fin coordination. Braking requires the robot to dissipate momentum without losing attitude stability. Hovering and station keeping require force production at near-zero forward speed, where normalized quantities based on *U* become singular or uninformative. Burst-and-coast operation may improve mission energy use even if instantaneous COT during the burst is high. These cases support the need for task-dependent metrics and separate reporting of steady and transient behavior.

The hydrodynamic environment also changes the effective gait. Currents alter the relative velocity seen by the body and fins, boundaries reshape pressure fields, and turbulence creates time-varying loading. A gait optimized in still water may not remain efficient or stable in a flow. Mechanism-level experiments should therefore identify the relevant nondimensional and structural ranges rather than report a single optimum as a universal setting.

Recent biological and computational evidence reinforces the need to treat stiffness as a time- and location-dependent descriptor. Lamprey measurements show that effective stiffness and damping depend on muscle-activation timing [34], while earlier physical models linked axial stiffness to swimming speed and waveform [35]. Self-propelled simulations further demonstrate that body stiffness changes propulsion rather than acting as a passive material label [36]. Experiments with attached flexible plates similarly show that fin length and stiffness must be interpreted jointly [37].

### 2.4. Dynamic Matching Descriptors for Compliant Swimmers

The recurring phrase “stiffness–frequency matching” is meaningful only after stiffness is defined. Material softness refers to a constitutive property, such as Young’s modulus, *E*; structural compliance depends on geometry as well as material and is commonly represented by a bending-rigidity distribution, EI(x)=E(x)I(x), or by an equivalent joint stiffness, $k_{j}$. Variable stiffness denotes the ability to change one of these structural quantities during or between operating conditions. These definitions should not be used interchangeably.

For a submerged body, the relevant dynamic reference is the wet natural frequency $f_{n,w}$ rather than an air measurement. A useful operating descriptor is the frequency ratio $r_{f}=f/f_{n,w}$, supplemented by the wet damping ratio $\zeta_{w}$, the phase lag between a command and realized posterior motion, Δϕ, and a tail-tip amplitude gain, such as $G_{A}=A_{tip}/A_{cmd}$. Added mass, fluid damping, payload, skin, wiring, and seals can shift all of these quantities. To make the previously qualitative Cauchy-type comparison explicit, this review defines

(1) $$q_{h}=\frac{1}{2}\rho U_{r}^{2},\;Ca_{EI}=\frac{q_{h}bL_{c}^{3}}{EI}=\frac{\rho U_{r}^{2}bL_{c}^{3}}{2EI},$$

where $U_{r}$ is the stated relative-flow scale, *b* is the loaded span or breadth, $L_{c}$ is the compliant length, and EI is the bending rigidity over the region being tested. For a caudal-fin-local analysis, $L_{c}$ should be the fin chord or compliant-fin span represented by the bending model; for a body-wave analysis, it should be the active flexible-body length. Free-stream speed is appropriate for steady translation, whereas hovering or oscillatory tests require a stated local velocity scale, such as 2πfA. Because alternative prefactors and plate-rigidity conventions exist, a reported Cauchy-type value is comparable only when $U_{r}$, *b*, $L_{c}$, EI, and the prefactor are all supplied.

**Figure 2:**
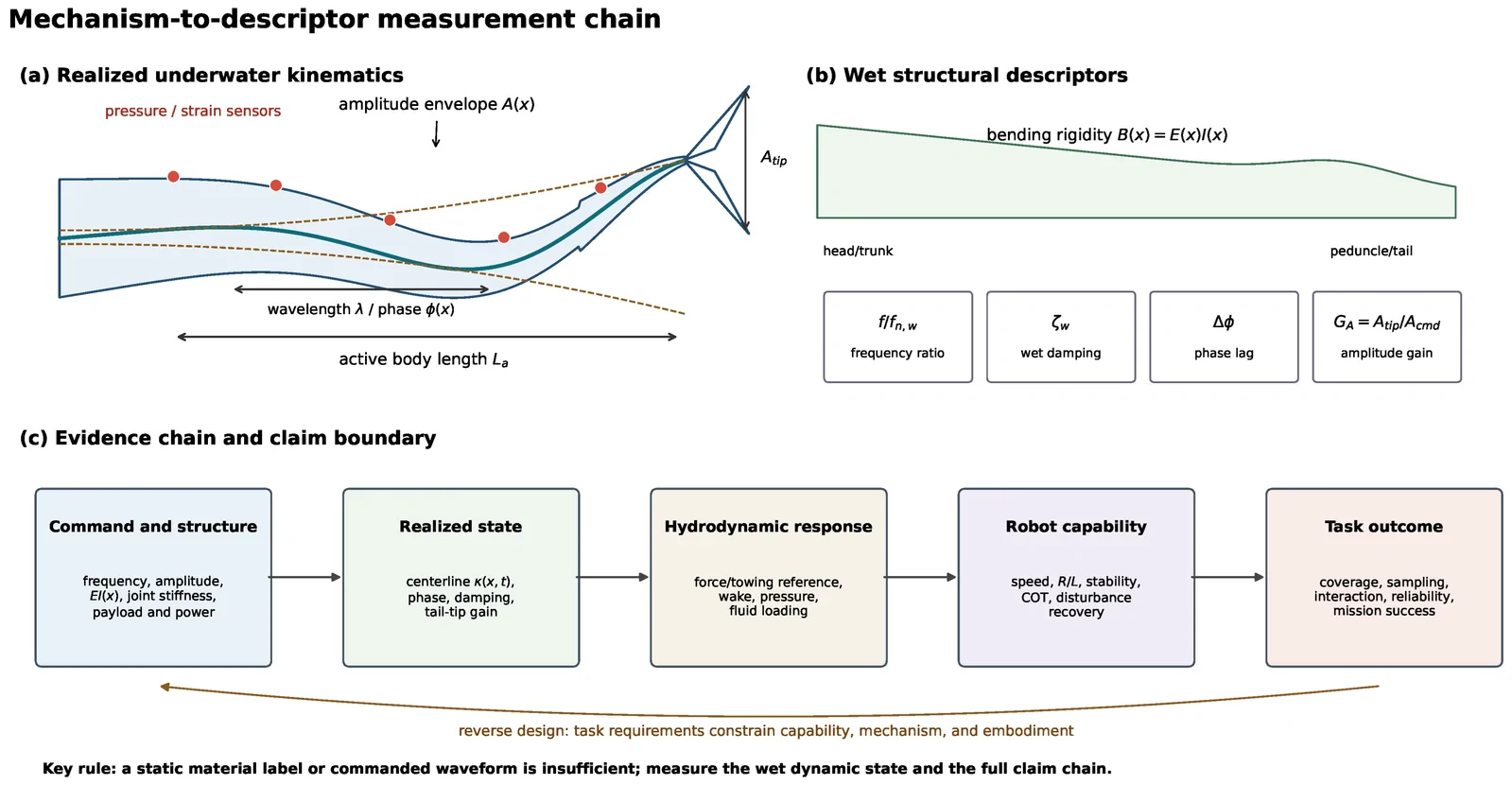
Mechanism-to-descriptor schematic for fish-like robot experiments. A traveling centerline is parameterized by active body length, amplitude envelope, wavelength, phase, and tail-tip motion; structural embodiment is represented by a wet bending-rigidity distribution, EI(x), damping, and a wet natural frequency; sensor locations define the observable pressure, strain, or flow field. The figure emphasizes the measurable chain from command and structure to realized curvature and phase, hydrodynamic response, robot capability, and task outcome.

The minimum causal chain for a dynamic-matching claim is therefore

$$EI\left(x\right),\;f/f_{n,w},\;\zeta_{w}\;⟶\;\kappa \left(x,t\right),\;\Delta \phi ,\;G_{A}\;⟶\;F_{T},\;P,\;U,\;\mathrm{COT},$$

where κ(x,t) is the realized centerline curvature. A static modulus or a visually smooth body wave does not establish the chain. Same-platform stiffness and frequency scans provide comparatively direct evidence, although coverage of payload, current, and durability remains limited [15,25,38,39,40]. Figure 2 summarizes the measurable quantities needed to turn a biological body-wave description into an engineering experiment.

### 2.5. From Biological Mechanism to Design Variable

Figure 2 summarizes the translation from swimming mode to measurable kinematic, structural, sensing, and task descriptors. The figure intentionally avoids a one-to-one mapping. Anguilliform and carangiform robots can both exploit traveling waves; a thunniform robot may still use pectoral fins for maneuvering; and the same stiffness gradient may support different tasks at different frequencies.

The first group of descriptors concerns morphology: body length, depth, width, cross-sectional shape, caudal-peduncle geometry, fin area, aspect ratio, fin placement, buoyancy distribution, and mass distribution. These variables determine drag, stability, available volume, and the mechanical path through which actuator forces reach the water. Morphological similarity to a species is less important than identifying which dimension is expected to alter performance.

The second group concerns kinematics: frequency, amplitude envelope, wavelength, phase lag, curvature, tail-tip trajectory, fin stroke plane, and angle of attack. A compact parameterization is useful for control, but it must be linked to measured body motion. In flexible robots, commanded and realized amplitudes should be reported separately.

The third group concerns mechanics: joint stiffness, body stiffness distribution, fin stiffness, damping, recovery time, hysteresis, resonance, and variable-stiffness range. These descriptors determine whether passive dynamics store useful elastic energy or merely add phase lag and loss. Tunabot Flex provides a clear example: increasing flexibility improved average self-propelled speed and reduced the minimum cost of transport within the tested configurations [15]. This result supports a qualified rule—flexibility can improve high-performance swimming when it is matched to frequency and geometry—rather than the broader claim that softer bodies are always better.

The fourth group concerns sensing and control: sensor type, placement, bandwidth, calibration, delay, state estimator, controller update rate, and onboard versus external computation. These descriptors become part of morphology because the body surface and gait determine the pressure and strain fields available to the sensor. A lateral-line-inspired array is not fully characterized by sensor count alone; body position and self-motion determine what pressure pattern reaches the array. Sensor placement optimization and self-motion compensation are therefore part of structural design rather than afterthoughts [41,42].

#### 2.5.1. Descriptor Coupling and Identifiability

Descriptors are useful only if they can be measured independently enough to support interpretation. In practice, geometry, stiffness, and kinematics are coupled. Increasing fin thickness changes stiffness and mass, adding a sensor changes surface geometry and wiring, moving a battery changes inertia and buoyancy, and changing frequency can alter amplitude because of actuator saturation or resonance. A well-designed experiment, therefore, distinguishes controlled inputs from realized states. Commanded frequency is an input, whereas measured tail-tip amplitude and body curvature are outcomes. Nominal material modulus is a fabrication property, whereas effective in-water stiffness is a system property.

This distinction is particularly important when deriving normalized quantities. BL/s requires a clear body-length convention and a representative speed. The Strouhal number requires a clearly defined amplitude. COT requires a power boundary and a steady or mission-averaged speed. When these conventions differ, reporting a normalized number with several decimal places creates false precision. The preferred practice is to retain the underlying dimensional variables and state the derivation explicitly.

#### 2.5.2. Biological Descriptors as Priors, Not Templates

Biological measurements can constrain a plausible design region, but robotic objectives may justify departures from the animal. A robot may require a larger rigid head for payload, a lower frequency because of actuator bandwidth, or an enlarged fin for low-speed control. The goal is not to preserve every biological proportion; it is to preserve the functional relationship needed for the task. Biological data are therefore best treated as priors for geometry, wave distribution, and timing, followed by controlled robotic optimization within engineering constraints.

A descriptor-based language also supports cross-scale reasoning. Geometrically similar robots at different sizes do not necessarily preserve dynamic similarity because actuator torque, material thickness, Reynolds number, and structural natural frequency scale differently. Scalable platforms such as ScaFi are valuable because they make the scaling assumptions explicit and allow the same parametric design logic to be tested across lengths [43]. Such evidence is stronger than separately optimized prototypes because it directly probes transferability.

Paired-fin evidence also spans several distinct mechanisms. Flexible bionic bones alter pectoral-fin hydrodynamics [44]; three-dimensional soft fins enable coupled stroke and feathering [45]; and pectoral-only swimming demonstrates that low-speed propulsion can be implemented without a caudal-dominant body wave [46]. These results support describing fin geometry, structural support, and stroke schedule separately rather than assigning a single MPF label.

### 2.6. Dimensionless and Normalized Quantities

Normalized quantities are necessary because robotic fish vary greatly in length and operating regime. The Reynolds number,

(2) $$Re=\frac{\rho UL}{\mu },$$

relates inertia and viscosity. Strouhal number,

(3) $$St=\frac{fA}{U},$$

relates oscillation frequency, *f*, a stated lateral amplitude, *A*, and swimming speed *U*. Body lengths per second, U/L, provides an intuitive normalized speed. The cost of transport is commonly represented as the gravity-normalized convention

(4) $$COT_{g}=\frac{P_{sys}}{mgU},$$

where $P_{sys}$ is the average power over a declared system boundary. For a neutrally buoyant vehicle, however, mg is not the useful propulsive force. Two complementary metrics are therefore recommended:

(5) $$E_{d}=\frac{P_{sys}}{U},\;COT_{D}=\frac{P_{sys}}{D_{tow}\left(U\right)U},$$

where $E_{d}$ is energy per traveled distance, and $D_{tow}\left(U\right)$ is the resistance measured by towing the same non-propulsive configuration at the same speed. $COT_{D}$ is the inverse of a quasi-propulsive-efficiency-type quantity only when the towing and powered-swimming boundaries match. The review retains published $COT_{g}$ values when that is the source convention but recommends reporting $P_{sys}$, $E_{d}$, and, where a matched towing test exists, $COT_{D}$ alongside it. Values are not back-calculated from motor nameplate ratings, battery capacity, or unmatched drag models. For electrically powered platforms, the preferred whole-system measurement is the synchronized bus voltage and current acquired using synchronized DAQ hardware or an equivalently calibrated acquisition system,

(6) $$P_{bus}\left(t\right)=V_{bus}\left(t\right)I_{bus}\left(t\right),\;{\bar{P}}_{sys}=\frac{1}{T}\int_{t_{0}}^{t_{0}+T}P_{bus}\left(t\right)\;dt,$$

with the sampling rate or bandwidth, sensor calibration, averaging window, and included loads being stated explicitly. Drivers, pumps, valves, onboard computation, sensors, communication, and lighting should be included or excluded by declaration rather than implicitly omitted.

These measures are only comparable when definitions are explicit. Tail amplitude may mean one-sided or peak-to-peak displacement. Body length may include or exclude the caudal fin. Power may omit pumps, computation, lighting, or tether losses. Water properties also matter across facilities and field sites because ρ and μ vary with temperature, salinity, and pressure; these variables shift both Re and the matched towing resistance $D_{tow}\left(U\right)$. Temperature and salinity should therefore accompany cross-site hydrodynamic or towing comparisons. By contrast, a towing reference measured in the same water and under the same conditions inherently incorporates the local fluid properties. A normalized value without protocol context can therefore create false precision. The review uses normalized data primarily to interpret trends within a study and only cautiously across platforms.

Turning performance presents a similar problem. Absolute radius favors small robots, whereas R/L supports scale comparison. The yaw rate captures rotational speed but not path accuracy or post-turn stability. A complete maneuvering report should state command, initial speed, turn direction, depth, tank dimensions, trajectory-extraction method, and repeated-trial variability.

#### Efficiency and Thrust Boundaries

For a tethered foil or tail, mean thrust and mechanical input can be defined directly within the rig. For a self-propelled swimmer at steady speed, net time-averaged force is approximately zero, so “thrust” generally requires a decomposition, a tethered surrogate, or a towing reference. Froude-type propulsive efficiency, quasi-propulsive efficiency, actuator mechanical efficiency, electrical drive efficiency, and whole-system efficiency answer different questions. Likewise, transport metrics should identify mechanical, electrical-drive, or whole-system boundaries. The gravity-normalized $COT_{g}$ is retained as a literature convention, whereas $E_{d}$ communicates mission energy per distance and $COT_{D}$ compares power with a matched useful towing reference. None should be inferred when the power boundary or towing configuration is incomplete. These distinctions prevent a favorable tail-rig result from being interpreted as complete-robot endurance.

### 2.7. Integrated Mechanism Examples and Experimental Interpretation

The mechanism-to-descriptor mapping becomes most useful when several variables are interpreted together. Consider a tail-dominant swimmer that reaches a high normalized speed. The result may arise from a favorable caudal-fin wake, from an actuator capable of sustaining high frequency, from a narrow operating resonance, or from a low-drag test configuration. Without measured body kinematics, power, and protocol context, these explanations cannot be separated. A mechanism-based interpretation therefore follows a chain: identify the input, measure the realized body and fin motion, characterize the fluid or force response, and then connect that response to free-swimming performance.

The Tunabot experiments illustrate this sequence. The platform was designed to access high tail-beat frequencies and to provide repeatable kinematic and hydrodynamic measurements [14]. Tunabot Flex then changed body flexibility within a related architecture and reported changes in speed and cost of transport [15]. The stronger conclusion is not that all flexible fish are faster. It is that, for the tested morphology and operating range, body flexibility modified the realized kinematics and improved selected performance outcomes. Transfer to another scale or actuator requires a new stiffness–frequency characterization.

A second example is paired-fin compliance. A rigid commanded stroke does not uniquely define the hydrodynamic fin motion because the membrane and joint can feather under load. Passive feathering may maintain a useful angle of attack and reduce the need for active pitch control [20]. Yet too much compliance can collapse the fin or delay force generation. The required evidence includes fin trajectory, deformation, force direction, and whole-robot motion under a common stroke protocol. Comparing only the material modulus or maximum deflection cannot establish a maneuvering advantage.

Flow sensing provides a third example. A pressure array can detect a flow feature under stationary calibration, but the useful robotic mechanism is the transformation from distributed pressure to an estimated variable that improves behavior. Closed-loop speed control, wall following, and online state estimation provide progressively stronger links from sensor to task [47,48,49]. Their limitations also differ: speed estimation depends on gait and flow calibration; wall following depends on boundary geometry; and state estimation depends on the observability of the chosen sensor layout. A single label, such as “artificial lateral line,” therefore hides several distinct mechanisms.

These examples support a common experimental sequence. The proposed causal pathway should be stated first, followed by the descriptor to be perturbed and the variables that may confound it. Experiments should then measure the realized structural and kinematic state, compare the intervention with a controlled baseline, and identify the operating conditions under which the effect weakens, disappears, or reverses. A mechanism can remain useful within a narrow range, but that range becomes part of the principle’s validity domain.

The template also helps reconcile biological fidelity and engineering optimization. A robot may deliberately deviate from biological geometry while preserving the relevant phase relationship, compliance distribution, or sensing principle. Conversely, a visually accurate body may fail to reproduce the functional coupling. The quality of biomimicry is therefore assessed by whether the targeted mechanism is represented and experimentally supported, not by the number of anatomical details copied.

### 2.8. Mechanism Synthesis and Remaining Contradictions

Across these mechanisms, the strongest conclusions are conditional on the realized in-water state. Deformation distribution should be specified before actuator selection; wet stiffness and damping should be treated as operating variables; commanded and realized kinematics should be distinguished; and wake observations should be interpreted together with power and whole-robot outcomes. Apparent contradictions often arise because similar labels conceal different stiffness distributions, Reynolds numbers, amplitudes, and power boundaries. These recurring findings are carried forward as conditional principles with explicit supporting and limiting evidence.

## 3. Morphology, Actuation, and Compliance Co-Design

### 3.1. A Continuum Rather than a Rigid–Soft Binary

**Figure 3:**
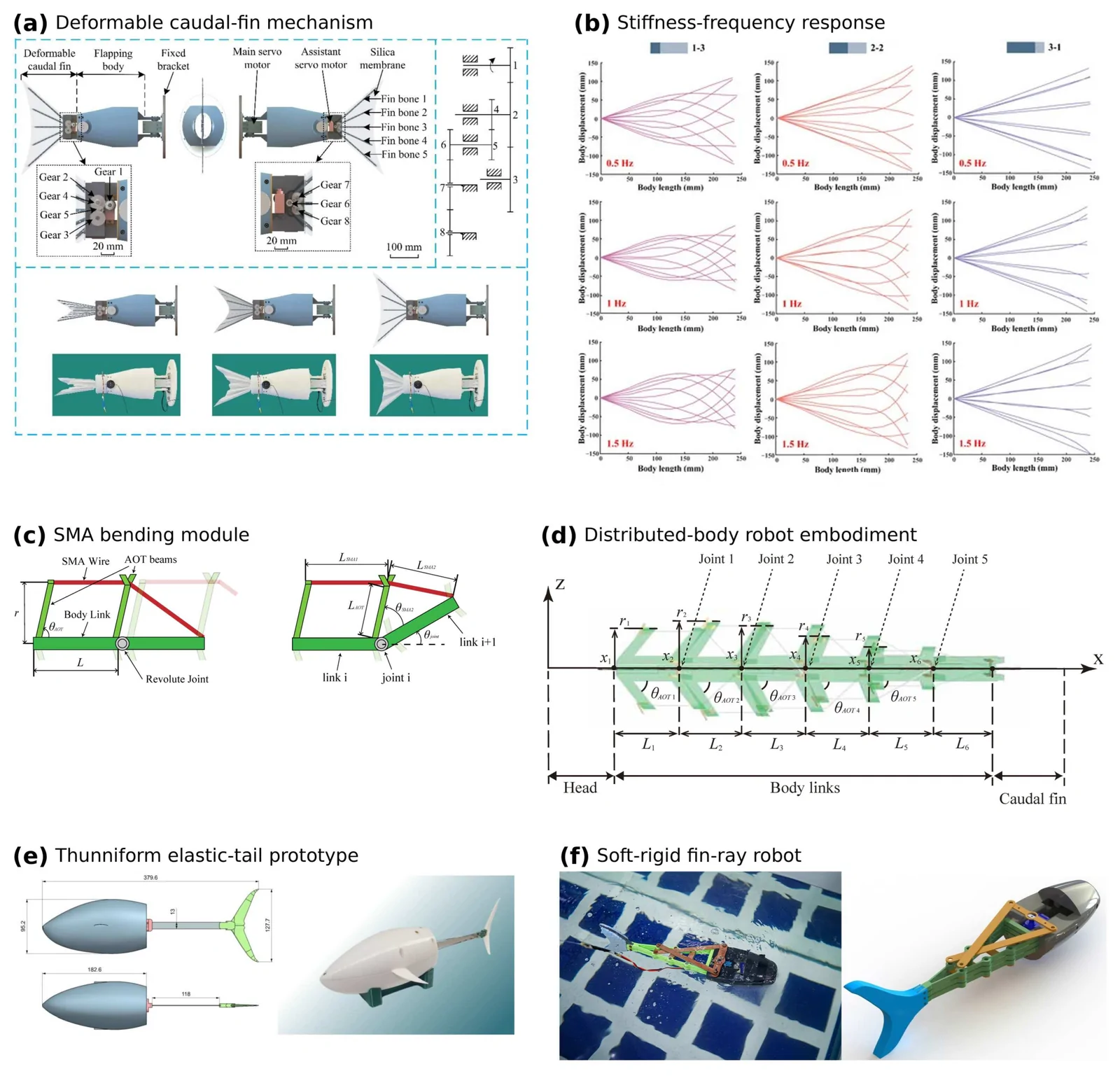
Representative examples of morphology, actuation, and compliance in fish-like robots. (**a**) Deformable caudal-fin design and prototype, adapted from Shao et al. [50], Figure 1(b–d), under CC BY 4.0. (**b**) Frequency-dependent displacement envelopes for non-uniform-stiffness foils, adapted from Zheng et al. [51], Figure 10, CC BY 4.0. (**c**,**d**) SMA-driven bending module and distributed-body UEC Mackerel embodiment, adapted from Aragaki et al. [52], Figures 2 and 4, CC BY 4.0. (**e**) Thunniform elastic-tail design and prototype, adapted from Mitin et al. [53], Figure 1, CC BY 4.0. (**f**) Soft–rigid fin-ray Pangasius robot, adapted from Youssef et al. [54], Figure 1, CC BY 4.0. Panels were cropped, resized, and relabeled for composition; inherited source-panel sequencing labels were removed. Scientific content was otherwise unchanged.

Morphology is an active component of propulsion, sensing, and control. The body transmits actuator forces, stores and dissipates energy, limits curvature, shapes the wake, supports payload, and determines how the robot responds to collision, pressure, and manufacturing variation. Actuation is equally inseparable from structure: useful output depends on stiffness, damping, fin geometry, water loading, sealing, power conversion, and the required operating frequency. Figure 3 presents representative examples spanning deformable fins, stiffness-dependent response, distributed actuation, and complete robotic embodiments. The panels are not a performance ranking; rather, they show how distinct mechanisms are physically realized and tested under different evidence contexts.

The visual comparison in Figure 3 highlights three recurring embodiment choices. Panels (a,b) isolate how prescribed deformation and stiffness distribution alter the response of a propulsive surface; panels (c,d) show how distributed actuation can be embedded along the body; and panels (e,f) illustrate complete tail-dominant and soft–rigid embodiments. Read together, the panels support the central argument of this section: morphology, actuation, and compliance are most meaningfully evaluated as a coupled physical system rather than as independent component categories.

Rigid multi-joint systems provide high command authority, repeatable kinematics, and convenient integration of motors, batteries, electronics, and pressure housings. Their finite links simplify modeling but introduce curvature discontinuities, concentrated reaction loads, and multiple sealing interfaces. Rigid–soft hybrids retain a load-bearing head, backbone, or transmission while adding a flexible posterior body, skin, or fin. Fully soft systems integrate deformation into the body and can provide continuous curvature and safe contact, although state estimation, payload support, and actuator power density are often more difficult. Variable-stiffness systems add the mechanical response itself as a controllable state. These categories overlap: a variable-stiffness mechanism may be embedded in a rigid, hybrid, or soft platform, and a visually soft exterior may conceal a mechanically rigid internal structure.

The representative evidence discussed in the main text spans mechanically coordinated, soft, hydraulic, piezoelectric, tendon-driven, tensegrity, and bistable systems. Spatially oscillating caudal-fin prototypes illustrate low-DOF mechanical coordination [55]. Transformable tails explicitly couple geometry to operating state [56]. Wire-driven compliant bodies provide measured straight and turning data while keeping motors away from the deforming posterior body [57]. A biomimetic red-muscle/tendon/vertebra mechanism provides another route to distributed actuation [52].

In the intervention evidence summary, “NR” is used when a value cannot be traced unambiguously. Missing whole-system power, payload, repeatability, or durability is not a minor bibliographic inconvenience; it limits the claims that can be made from otherwise impressive demonstrations.

### 3.2. Rigid Multi-Joint and Mechanically Coordinated Architectures

Rigid robotic fish established a practical bridge between fish kinematics and mechatronic implementation. Serial joints driven with phase-shifted commands made body waves programmable and created repeatable platforms for control and hydrodynamic experiments [9,11]. Their continuing strength is observability: commanded angles, measured joint states, and body configuration can be related directly, which supports model identification, controller tuning, and controlled gait sweeps. A rigid architecture is therefore not merely an early stage that is superseded by softness; it remains advantageous as an experimental platform when precise kinematic control and repeatability are prioritized over continuous deformation.

The number and placement of joints define both capability and burden. More actively controlled segments can approximate a continuous curvature profile and enlarge the gait space, but they increase wiring, waterproof penetrations, calibration, and control dimensionality. Low-degree-of-freedom designs deliberately accept a restricted body wave in exchange for compactness and robustness. Ostraciiform platforms, including segmented caudal-fin designs, show how tailoring the tail structure can recover part of the lost hydrodynamic flexibility without multiplying active joints [10,19].

Mechanically coordinated and underactuated designs reduce actuator count through linkages, elastic joints, tendons, magnetic transmissions, or shared drives. The advantage is not free simplicity: control authority is exchanged for dependence on mechanism geometry, stiffness, damping, and fluid load. A passive joint can smooth the body wave at one frequency and become poorly phased at another. Consequently, an underactuated architecture is most interpretable when its operating envelope is defined and sensitivity to frequency, payload, and current is characterized. Reporting only the best gait obscures the mechanism’s robustness.

Tail-dominant thunniform systems illustrate the value of specialization. Concentrating deformation near the peduncle stabilizes the anterior body and permits high-frequency tail actuation, but performance depends on low-backlash transmission, body inertia, fin geometry, and dynamic stiffness. Tail-dominant robotic swimmers have demonstrated the value of integrated high-frequency design [58]. Tunabot reached high normalized speeds in a controlled flow facility and functioned as a repeatable physical model of fish-like propulsion [14]. The evidence is strong for the tested mechanism and protocol; it does not automatically establish untethered endurance, payload capacity, or open-water reliability.

Rigid structures also facilitate payload integration. Dry compartments can support cameras, batteries, processors, acoustic devices, and buoyancy hardware. The trade-off is mechanical: a long rigid compartment suppresses distributed deformation and raises rotational inertia. Tail-dominant designs accept this deliberately, whereas general-purpose subcarangiform robots often require a graded transition between the payload-bearing body and the propulsor. The design variable is therefore not simply “rigid body length” but the allocation of stiffness, mass, and actuation authority along the robot.

### 3.3. Rigid–Soft Hybrids and Passive Compliance

Hybrid architectures address a common system requirement: the robot must carry rigid components while generating a smooth, load-adaptive propulsive wave. A rigid head or backbone can house payload and reaction forces, while a compliant tail, skin, or fin participates in fluid–structure interaction. The critical region is the interface. An abrupt stiffness change can localize curvature, concentrate stress, accelerate fatigue, or create a leakage path. Graded-compliance layers, tapered transitions, stress-relief fillets, distributed elastic elements, flexible skins, and carefully routed tendons can reduce local strain concentration and smooth the transfer of bending moment. These features should be evaluated under wet cyclic loading because an interface that is benign in a dry static test may still delaminate or leak after repeated propulsion cycles.

Passive compliance can store and release elastic energy, provide beneficial phase lag, and allow the propulsor to adapt to water loading without an additional controller. Its effect is conditional rather than intrinsically positive. Tunabot Flex provided unusually direct evidence by changing body flexibility within a related platform and evaluating both speed and transport cost [15]. Computational and robotic studies similarly show that stiffness changes internal bending moments, fluid reaction, and posterior phase [25,26]. The evidence supports the conclusion that compliance can improve performance when its spatial distribution and dynamic response are matched to forcing frequency, geometry, and load.

Passive fin compliance deserves separate treatment. Pectoral and caudal fins may feather, twist, or alter camber under hydrodynamic loading. Such deformation can maintain a useful force direction and reduce the need for actively controlled pitch, but excessive compliance can collapse the fin, delay force generation, or reduce repeatability. Flexible-joint and folding-fin studies show that meaningful interpretation requires synchronized measurements of prescribed stroke, realized fin shape, force, and whole-robot motion [20,21]. Material modulus or maximum tip deflection alone is insufficient.

Hybrid systems can also improve maintainability, while multi-flexible architectures illustrate how distributed passive motion can be optimized jointly with energy use [59]. Energy-oriented optimization and power-cost analyses further show why actuator output must be evaluated together with transmission, electronics, adjustment mechanisms, and mission duration rather than as an isolated efficiency claim [60,61]. Electronics and batteries may remain in replaceable sealed modules while skins, tendons, tails, or fins are serviced separately. This advantage is seldom reflected in maximum speed, yet it can determine whether a platform is viable for repeated experiments or field work. Useful engineering reports would therefore include assembly procedure, tendon pretension, interface fatigue, seal replacement, and recalibration after component exchange.

### 3.4. Fully Soft Fish-like Robots

Soft fish-like robots seek to generate body deformation through compliant materials, fluidic chambers, dielectric elastomers, shape-memory alloys, ionic polymer–metal composites, or other distributed actuators. Their central benefit is not softness by itself but the ability to create continuous curvature, tolerate contact, and integrate body and actuator functions. SoFi demonstrated the system-level relevance of this approach by combining soft propulsion, depth control, acoustic commands, and onboard imaging in marine observations [62]. The platform’s significance lies in matching architecture to a low-disturbance observation task rather than in maximizing speed.

Soft fluidic actuation can produce smooth, large-amplitude bending while isolating the surrounding water from exposed rigid mechanisms, as emphasized in broader reviews of underwater soft robotics [8,63]. A recent materials-focused review of stimulus-responsive soft actuators similarly shows that robotic performance depends on material–structure–interface coupling, energy-conversion efficiency, environmental adaptability, and long-term interfacial stability, not on free strain alone [64]. A recent review of magnetic soft robots is used here only as a cross-domain analogue for programmable deformation and remote actuation because its primary evidence base is biomedical [65]. For aquatic robots, the relevant actuation boundary may also include pumps, valves, reservoirs, pressure regulators, tubing, magnetic-field hardware, high-voltage electronics, and rigid control hardware. These elements alter mass, power demand, acoustic output, and reliability. Component-level curvature therefore becomes evidence of an untethered-robot advantage only when the support and power hardware required to produce it is included in the system boundary. Nonlinear input–curvature relations, hysteresis, leakage, field-generation cost, and fabrication variability further constrain open-loop control [66,67].

Electroactive and thermally activated materials offer alternative trade-offs. Ionic polymer–metal composites bend at low voltage in water and are attractive for miniature fins, but force output, hydration state, electrode durability, and drift constrain whole-robot use [68,69]. Shape-memory alloys provide compact high-force actuation but are limited by thermal cycling, efficiency, and cooling rate [70,71]. Dielectric elastomer actuators can respond rapidly and support highly compliant bodies, yet high electric fields and encapsulation requirements must be included in the safety and power assessment [72]. Piezoelectric actuation becomes attractive at small scales where compact high-frequency motion is valuable, but stroke amplification, resonance, and payload constraints remain central [73,74].

The term “fully soft” can be misleading if used only to describe the external material. Batteries, cameras, electronics, fittings, and valves often remain rigid. A more useful description states which regions carry load, which generate motion, and which are compliant under the relevant water pressure and frequency. Similarly, claims of safe interaction require measured contact force, failure mode, or behavioral response rather than inference from a soft skin alone.

Soft bodies create distinct sensing and modeling challenges. Strain, curvature, and chamber pressure may provide rich proprioceptive information, but embedded conductors and sensors must survive repeated bending and remain waterproof. Manufacturing variability means that two nominally identical bodies may require different calibration. Soft sensing and closed-loop control are therefore important not only for autonomy but also for compensating specimen-specific mechanics [75].

### 3.5. Variable Stiffness and Morphological Intelligence

Variable stiffness extends passive compliance by allowing the mechanical response to change with the operating conditions. The rationale is that transit, turning, hovering, and disturbance rejection may favor different deformation patterns. Stiffness can be varied through antagonistic structures, prestress, jamming, phase-change materials, tensegrity, adjustable springs, or actively controlled soft composites. Recent programmable mechanisms further show that stiffness can be modulated online rather than only between experiments [76]. Elastic-spine and integrated variable-stiffness mechanisms extend the same objective through different transmission and packaging choices [77,78].

The main design question is whether the gain from mechanical adaptation exceeds its cost. A stiffness-adjustment mechanism adds mass, volume, energy use, sensing demand, and possible failure modes. Its value should therefore be evaluated against at least two baselines: a fixed-stiffness body optimized for one condition and a fixed compromise body operated across the full task envelope. The relevant outcomes include not only the best speed or efficiency, but transition time, repeatability, actuator or pump energy, stability during reconfiguration, fatigue, leakage or hysteresis, and mission success. Existing studies establish condition-dependent locomotor benefits and attainable stiffness ranges more clearly than they establish net whole-system energetic benefit; complete adjustment-energy and reliability accounting remains sparse, so evidence for a net whole-system mission benefit remains provisional.

Reconfigurable platforms are particularly valuable as physical models because they enable within-system comparisons. The multimodal modular soft robotic fish demonstrated multiple swimming gaits on a common reconfigurable body [16]. ScaFi investigated a parametric compliant design across a broad length range [43]. These studies strengthen design inference because geometry, instrumentation, and the measurement boundary can be held more consistently than in comparisons among unrelated robots.

“Morphological intelligence” is useful only when operationally defined. A body can be said to contribute to control when its mechanics reduce actuator effort, sensing requirements, computation, or disturbance response relative to a stated baseline. Passive phase coordination, mechanical filtering, self-feathering fins, and stiffness-dependent gait selection are plausible examples. Without a measurable baseline, however, the term risks becoming a rhetorical synonym for compliance. High-frequency tensegrity and bistable-tail systems further illustrate that mechanical range and gait switching should be separated from demonstrated energetic benefit [79,80]. Nonlinear snapping or adjustable bistability can improve selected maneuvers, but only within a stated potential-well and actuation regime [81,82].

### 3.6. Actuator Selection as a Matched Design Problem

Actuators differ in torque or force density, bandwidth, stroke, efficiency, controllability, voltage, packaging, and compatibility with water. These properties cannot be ranked independently of the body and task. Motors and servos provide mature control and high authority but require transmissions, shafts, seals, or cable routing. Tendon and wire drives can relocate motors into a dry compartment and generate smooth body deformation, although friction, pretension, routing, and fatigue become critical. Linkages can mechanically synthesize a desired trajectory but may restrict gait adaptability.

Smart materials trade transmission complexity for material and driver complexity. Shape-memory alloys, ionic polymer–metal composites, dielectric elastomers, macro-fiber composites, artificial muscles, and piezoelectric actuators occupy different scale and frequency regimes. Recent examples include an SMA carangiform tail, DEA and MFC dynamic models, muscle-like flexible-tail actuation, and modular artificial-muscle bodies [83,84,85]. Flexible-tail and artificial-muscle studies further emphasize deformation control and whole-body integration rather than free strain alone [86,87]. Comparing their maximum free strain is rarely informative for a robotic fish. An informative comparison includes blocked force or torque, operating frequency in water, required driver, thermal or electrical efficiency, wet-state durability, achievable body curvature, and the mass of the complete actuation system.

More broadly, data-driven multi-objective material selection may provide a useful methodology for balancing wet stiffness, fatigue resistance, density, manufacturability, interface durability, and actuator compatibility. As a cross-domain methodological example, Xu et al. used machine-learning-guided optimization to balance fracture toughness and high-temperature strength in Nb–Si alloys [88]. This example illustrates the optimization strategy rather than direct aquatic-robot material evidence; transfer to compliant robotic bodies requires revalidation under wet cyclic loading, robot-relevant density and stiffness constraints, and the intended actuation frequency.

Fin-specific actuation requires control of stroke, feathering, and sometimes spanwise curvature. MPF systems may need several degrees of freedom to generate thrust, side force, and pitching moment independently. Mechanical simplification of ray- or fish-inspired fins can be effective when it preserves the dominant force-generation mechanism [22,89]. Fin-ray flexural rigidity can substantially affect fin deformation and the direction, magnitude, and time course of propulsive forces, emphasizing that fin geometry and stiffness distribution belong to the actuation design rather than serving as passive appendages [90]. Table 4 summarizes representative intervention evidence and its applicability boundaries.

The newer literature also broadens the range of stiffness mechanisms. Tunable caudal peduncles can change speed without adding a second propulsive actuator [39]. Bistability can enable gait switching but does not guarantee an efficiency benefit across the entire operating envelope [94]. Snapping structures create impulsive jet flows and rapid maneuvers [95]. Layer-jamming tails offer reversible stiffness at the cost of pneumatic hardware [96]. Modular passive joints and compressible spines target replaceable, distributed adaptation [97,98].

Table 4 summarizes the intervention evidence without presenting a universal ranking. Untethered and double-joint hydraulic tuna platforms also demonstrate that hydraulic authority can support integrated locomotion, while making pump, accumulator, sealing, and whole-system COT part of the comparison [99,100]. The appropriate selection follows from the required deformation, frequency, load, power source, sealing strategy, sensing arrangement, and maintenance interval. An actuator that performs well in air or in a tethered component test may be unsuitable after integration into a neutrally buoyant, untethered robot.

Pneumatic variable-stiffness caudal fins demonstrate that tracking performance and mechanics can be co-designed at the actuator level [101]. Their high-frequency limitation is best characterized from the realized wet-state transfer function rather than assigned a universal cutoff. Bandwidth characterization is more informative when it includes the amplitude gain $G_{A}\left(f\right)=A_{realized}\left(f\right)/A_{command}\left(f\right)$ and command-to-deformation phase lag Δϕ(f) across frequency, because chamber volume, tubing, valve flow, supply pressure, wall stiffness, and water loading jointly determine usable bandwidth. The transition from a propulsive traveling deformation to a weak or standing-wave response is therefore platform-specific; a fixed threshold such as 2 Hz is not transferable across fluidic architectures. Fluid-driven collapsible bodies show a different integration route for compact storage and deployment [102]. Liquid–vapor swim-bladder mechanisms illustrate how buoyancy and body mechanics can be coupled, but also expose thermal and pressure-management requirements [103].

### 3.7. Waterproofing, Manufacturing, Payload, and Lifecycle Integration

Waterproofing is part of the mechanical design, not a final enclosure step. Moving shafts and bearings introduce friction and leakage risk; tendon exits and flexible feedthroughs concentrate wear; bonded soft interfaces can delaminate; and thin membranes may be damaged by abrasion or puncture. Pressure compensation and deep-water operation add compressibility and buoyancy constraints. The selected sealing strategy can change actuator output and body stiffness, and must therefore be included in performance characterization.

Manufacturing variability is especially consequential for compliant systems. Small changes in wall thickness, cure ratio, fiber orientation, fin prestress, or tendon pretension alter the realized body wave. Nominal CAD geometry does not capture these effects. Useful characterization includes mass and buoyancy distribution, wet-state static and dynamic stiffness, actuator-to-curvature calibration, and variability across specimens. For modular platforms, the recalibration required after replacing a tail or fin is part of maintainability.

Payload integration creates a second coupling. Cameras, samplers, water-quality sensors, acoustic modems, and onboard computers modify mass, center of buoyancy, drag, and power demand. A robot evaluated without the payload required by its intended mission may overestimate locomotion and stability. Conversely, a properly placed payload can improve inertial stability even if it reduces acceleration. The operational configuration should be defined before finalizing stiffness and gait.

Durability remains underreported. Soft skins and fins may degrade through creep, fatigue, delamination, or puncture; tendons may stretch or fray; rigid transmissions may develop backlash, corrosion, or seal wear. Protective structures such as bio-inspired scale systems seek to improve damage tolerance without eliminating compliance [104]. Reliability assessment is strengthened by reporting cycle count, performance drift, inspection criteria, repair, and failure mode rather than presenting only successful short trials.

### 3.8. Scale, Pressure, and Environmental Durability

Scaling a fish-like robot is not a geometric exercise. Actuator torque or force, structural natural frequency, fin thickness, battery volume, surface area, and Reynolds number follow different scaling laws. A mechanism that is effective at tens of centimeters may be impractical at a few centimeters because driver and encapsulation volume dominate, or at several meters because bending loads and structural mass increase. Scalable parametric platforms are valuable because they expose these changes within a common design language rather than hiding them in independently optimized prototypes [43].

Miniature robots face particularly tight integration constraints. The energy source, controller, high-voltage converter, and sealing can occupy more volume than the propulsive element. Resonant piezoelectric or electroactive mechanisms may provide high-frequency motion, but the usable stroke and control range depend on water loading and manufacturing tolerance. The dominant fluid regime cannot be inferred from frequency alone: characteristic length, realized oscillation amplitude, fluid viscosity, and frequency jointly determine the balance between inertial and viscous effects. For an oscillatory velocity scale, $U_{\omega }=\omega A$, two useful descriptors are

(7) $$Re_{\omega }=\frac{\rho \omega AL_{c}}{\mu },\;\alpha_{o}=L_{c}\sqrt{\frac{\omega \rho }{\mu }}=L_{c}\sqrt{\frac{\omega }{\nu }},$$

where *A* is the realized one-sided oscillation amplitude, $L_{c}$ is the stated characteristic propulsive length, ω=2πf, and ν=μ/ρ. Here, $Re_{\omega }$ uses the realized oscillatory speed to compare inertial and viscous effects, whereas the Womersley/Stokes-type parameter $\alpha_{o}$ compares the oscillation time scale with viscous diffusion across $L_{c}$. Larger values indicate increasing importance of unsteady/inertial effects relative to viscous diffusion, while smaller values indicate stronger viscous penetration; neither quantity defines a universal transition threshold. Discussions of the force regime at millimeter and centimeter scales are therefore most interpretable when the chosen $L_{c}$, realized *A*, *f*, and fluid properties are reported together with these nondimensional descriptors. The payload capacity is correspondingly limited, so a high normalized speed should not be confused with general mission capability [73,105]. At larger scales, the challenge shifts toward structural loading, distributed actuation, and the power required to maintain the intended curvature field.

Depth introduces another set of couplings. External pressure changes buoyancy, compresses trapped volumes, loads seals, and can alter soft-material response. A pressure-tolerant body may use oil filling, pressure compensation, distributed electronics, or nearly incompressible soft materials, but each strategy affects stiffness and serviceability. Laboratory tests near the surface cannot support deep-water claims unless the structural and electrical pressure boundary has been validated. Conversely, a pressure-tolerant soft body may remain limited by connectors, cameras, batteries, or actuators that require separate housings.

Environmental durability is similarly mechanism-specific. Abrasion and puncture threaten exposed soft skins; sediment can enter moving joints; biofouling alters flow-sensor ports and fin compliance; temperature affects elastomers, batteries, and thermal actuators; and salt water accelerates corrosion and electrode degradation. Protective scale-like structures can improve damage resistance while retaining global compliance, illustrating that biological inspiration can target protection as well as propulsion [104]. The cost of protection—added drag, mass, stiffness, and manufacturing complexity—should be evaluated on the assembled robot when protection-related performance is claimed.

These scaling and environmental effects reinforce a broader design principle: the relevant design object is the complete robot in its wet, powered, payload-carrying configuration at the intended depth and mission duration. Dry material data and unloaded tank kinematics remain necessary inputs, but they do not define the operational structure. Reporting the environmental envelope helps distinguish a mechanism demonstrator from an architecture that has been validated for repeated deployment.

### 3.9. Task-Conditioned System Archetypes

The architecture continuum yields several recurring task archetypes. A high-speed physical model favors a rigid payload body, low-backlash transmission, well-characterized tail flexibility, and extensive kinematic and hydrodynamic instrumentation. A low-disturbance observation robot favors a smooth exterior, contact safety, stable imaging, and a compliant propulsor, while retaining sufficient rigid volume for energy and computation. An inspection or sampling platform often benefits from a hybrid body: caudal propulsion supports transit, auxiliary fins support local control, and a rigid compartment supports payload and recovery. A miniature swimmer may use very few active degrees of freedom because driver, battery, and sealing volume dominate the available scale. A reconfigurable physical model prioritizes controlled variability in stiffness, activation length, or fin geometry over optimization for one mission.

These archetypes clarify the intended contribution of a platform. A mechanism model is evaluated by controlled perturbation and explanatory measurement. A capability demonstrator is evaluated by integrated locomotion or closed-loop performance. A mission prototype is evaluated by task outcomes, operational payload, failures, and recovery. Ambiguity among these roles frequently leads to inappropriate comparison: a highly instrumented flow-tank model may provide stronger hydrodynamic evidence than a field robot, while the field robot provides stronger deployment evidence.

### 3.10. Structural Synthesis

Rigid, hybrid, soft, and variable-stiffness platforms occupy overlapping regions of a common design space. Across the studies reviewed here, spatial stiffness, wet dynamic response, system mass, and actuation/support hardware provide more transferable information than qualitative labels such as “soft”. No single architecture is superior across all tasks. Section 4 therefore treats sensing and control as embodied components of this design space, while the concluding synthesis consolidates the structural conclusions with their evidence limits and discriminating validation tests.

## 4. Sensing, Control, and Embodied Autonomy

### 4.1. From Prescribed Gait to Closed-Loop Behavior

**Figure 4:**
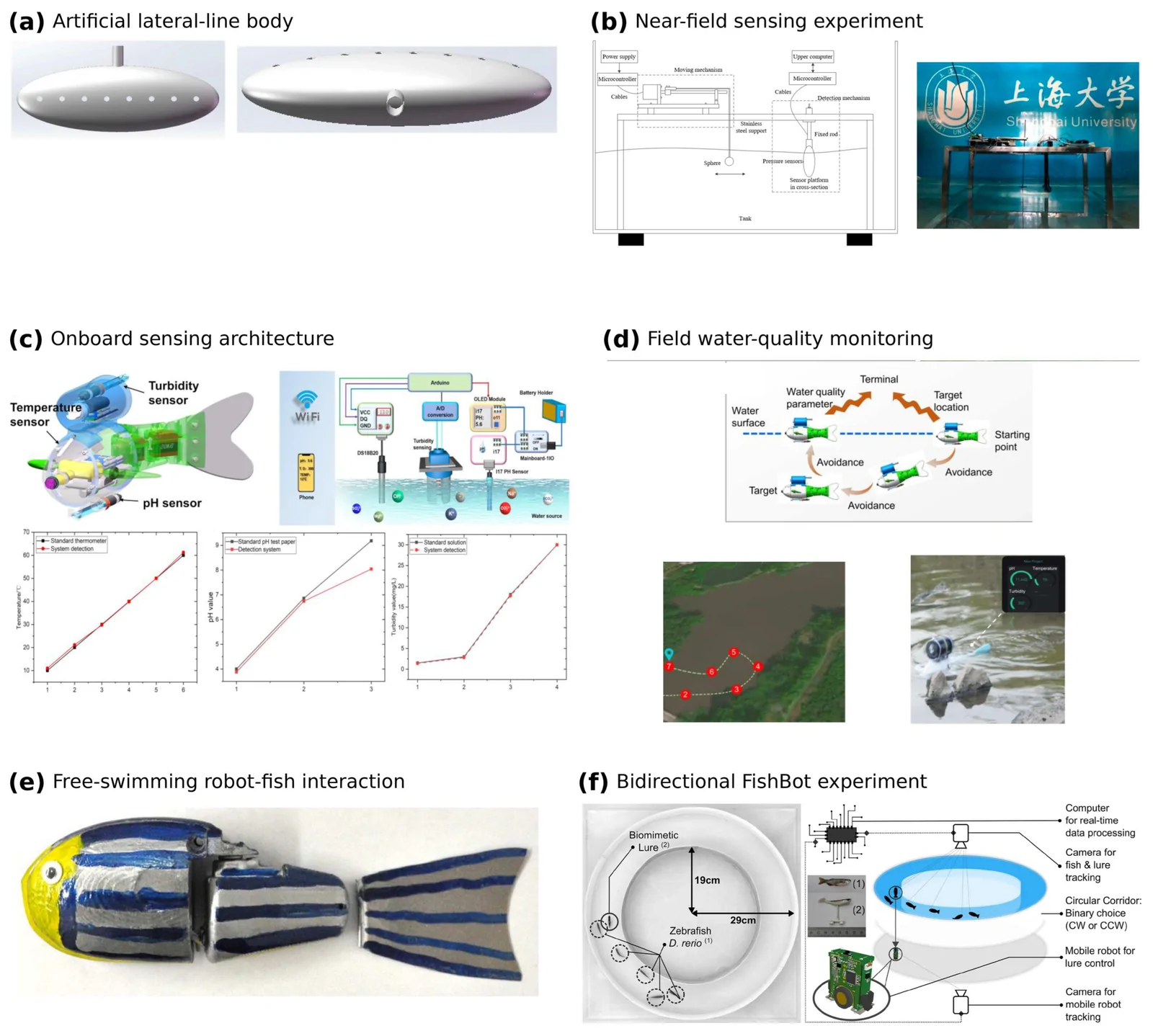
Representative examples of sensing, closed-loop experimentation, and task validation. (**a**,**b**) Fish-shaped artificial lateral-line body and near-field detection setup, adapted from Tang et al. [106], Figures 3 and 4, under CC BY 4.0. (**c**,**d**) Onboard water-quality sensing/network architecture and on-site monitoring demonstration, adapted from Chen et al. [107], Figures 6 and 7, CC BY 4.0. An identifiable non-data photograph in the source material for panel (**d**) was omitted for privacy; the system schematic, route map, and robot-only field image were retained. (**e**) Free-swimming robotic fish used in zebrafish-shoal experiments, adapted from Butail et al. [108], Figure 1, under the Creative Commons Attribution license stated on the official article page. (**f**) Circular-corridor FishBot experiment supporting bidirectional animal–robot interaction, adapted from Papaspyros et al. [109], Figure 1, CC BY 4.0. Panels were cropped, resized, and relabeled for composition; inherited source-panel sequencing labels were removed. Scientific content was otherwise unchanged.

Task-level autonomy generally requires a closed sensing–estimation–control loop in which robot state or environmental information influences subsequent behavior. Many platforms can execute a programmed oscillation, but this alone does not establish perception, adaptation, or robustness. The relevant autonomy question is whether the robot estimates a state or environmental variable and uses that information to improve behavior under disturbances, uncertainty, or task changes. Figure 4 grounds the sensing–decision–action loop in representative hardware and task evidence, from artificial lateral-line instrumentation to field monitoring and animal–robot interaction. The panels deliberately distinguish sensing hardware, controlled test infrastructure, integrated task systems, and biological-interaction experiments.

Figure 4 is organized from sensing hardware to integrated use. Panels (a,b) isolate artificial-lateral-line embodiment and controlled near-field testing; panels (c,d) connect onboard sensing to field water-quality monitoring; and panels (e,f) extend the evidence boundary to animal–robot interaction. This progression clarifies that sensor response, closed-loop control, field task execution, and biological acceptance are distinct validation levels.

The architecture is embodied because morphology affects every layer, a coupling that also motivates recent reviews of fish-robot modeling and control [110]. Body compliance determines how actuator commands become curvature; sensor placement determines which pressure or strain fields are observed; the gait creates self-generated flow that can mask environmental signals; and the controller can excite or suppress structural modes. For this reason, comparing control algorithms without reporting body state, sensing boundary, and disturbance conditions provides limited design insight.

Four functional configurations are useful for distinguishing the roles of feedback and perception. In the first, open-loop commands generate a repeatable gait. In the second, feedback regulates an internal or externally measured robot state, such as heading, depth, or speed. In the third, onboard perception estimates environmental features such as flow direction, boundaries, or obstacles. In the fourth, the robot selects or adapts behavior according to a task. These configurations are not a universal ordinal ranking: an open-loop physical model can be scientifically valuable, but it should not be described as autonomous navigation.

### 4.2. Proprioceptive and Exteroceptive Sensing

Proprioceptive sensing describes the robot’s own state. Common measurements include joint angle, actuator position or current, chamber pressure, tendon tension, strain, inertial motion, depth, and battery state. Rigid robots can often measure joint configuration directly, while compliant bodies require distributed or inferred curvature. The distinction matters because the commanded waveform may differ substantially from the realized body wave. Measuring deformation is therefore necessary both for control and for interpreting hydrodynamic experiments.

Exteroceptive sensing describes the surrounding environment. Cameras provide rich scene information but depend on lighting, turbidity, and computational resources. Acoustic sensing and communication support longer ranges but offer lower spatial resolution and may conflict with low-disturbance objectives. Pressure and flow sensors are attractive because they can detect local hydrodynamic features in darkness or turbidity. Artificial lateral-line systems have consequently become a distinctive research direction in robotic fish [23,42].

Sensor integration alters the body. Pressure ports, rigid sensor packages, cables, and protective coatings can disturb the surface or change local stiffness. In a soft robot, embedded strain sensors can constrain deformation or fail under repeated bending. The sensor layout must therefore be designed together with morphology, rather than selected solely by estimator performance. Placement optimization can reduce redundancy, but the optimization objective must correspond to the intended variable and operating gait [41].

#### 4.2.1. Separating Environmental Information from Self-Motion

A swimming robot generates pressure fluctuations through its own body and fin motion. The same sensor array may therefore observe current, wake, boundaries, and self-induced flow simultaneously. Calibration on a stationary body is insufficient for a moving platform. Useful approaches include gait-synchronous subtraction, model-based prediction of self-flow, multimodal fusion with inertial or actuator state, and training data that span the intended kinematics. The key test is not whether a signal is detectable in a static tank but whether the relevant environmental variable remains observable during realistic motion.

#### 4.2.2. Calibration, Drift, and Field Effects

Flow and pressure sensors are sensitive to mounting, temperature, bias, fouling, and surface damage. Soft strain sensors add hysteresis and rate dependence. Field-oriented reports should state calibration procedure, update rate, filtering, drift over time, and recalibration after maintenance. A sensor that performs well in short laboratory trials may require periodic cleaning or model adaptation in natural water. These effects are especially important when a controller depends on small pressure differences.

### 4.3. Artificial Lateral Lines and Flow-Aided Behavior

Artificial lateral-line studies span sensor characterization, flow reconstruction, object detection, state estimation, and closed-loop control. Hydrodynamic pressure arrays have been characterized in steady and unsteady flows, establishing the physical basis for distributed sensing [24]. Subsequent work used such measurements for distributed flow estimation, flow-relative control, speed regulation, wall following, and online state estimation [49,111,112]. These studies are valuable because they connect the sensing mechanism to behavior rather than stopping at signal classification. Table 5 summarizes representative sensing and closed-loop task evidence while preserving these distinct evidence scopes.

The strength of a flow-aided control result depends on its baseline. A controller should be compared with an equivalent controller without the flow measurement, or with a conventional sensing modality under the same disturbance. Reporting estimation error alone does not establish task benefit. For wall following, relevant outcomes include stand-off distance, trajectory error, speed range, geometry variation, and recovery after losing the boundary. For flow-relative control, relevant outcomes include heading or velocity relative to the water, current range, and sensitivity to gait.

Near-field object detection introduces another evidence boundary. A pressure pattern can indicate the presence of an object in controlled geometry [106]; field use additionally requires robustness to body motion, current, multiple boundaries, and changes in sensor surface condition. Likewise, a sensor-placement algorithm can reduce redundancy, but the mission value must still be established on the physical robot. The progression from sensor response to state estimate to closed-loop outcome should therefore remain explicit.

Recent systems make the autonomy boundary increasingly explicit. Multi-sensory recovery integrates binocular vision and infrared distance sensing into a four-stage physical recovery sequence [119]. Route-estimation work targets autonomous swimming without assuming that the desired path is already available [120]. Vision-based obstacle avoidance has been extended from single-robot navigation to formation maintenance [121]. Decentralized pursuit control applies reinforcement learning at the multi-robot task level rather than only to individual gait tuning [122]. A 2026 review of reinforcement-learning-enabled rigid-link robotic fish further shows that learning is now being used across gait generation, control, and design, while emphasizing continuing sim-to-real, state-observation, and validation challenges [123].

Table 5 summarizes representative evidence while preserving its scope. Sensor characterization, state estimation, and closed-loop task performance are complementary but not interchangeable. This distinction prevents an accurate offline classifier from being presented as autonomous capability.

#### Vision for Mission-Oriented Inspection

Navigation vision and inspection vision should also be distinguished. A detector can identify an obstacle without demonstrating the image quality, defect localization, or uncertainty needed for structural inspection. Recent underwater-inspection literature couples robotic data acquisition with enhancement, deep detection or segmentation, defect quantification, and three-dimensional localization, while also documenting the strong effects of turbidity, illumination, stand-off distance, and motion blur [124,125]. These results are transferable to fish-like inspection missions at the task-stack level rather than direct evidence that a fish-like morphology improves defect detection. A fish-like inspection platform should therefore report camera/lighting geometry, image-stability or blur metrics, inference location and latency, defect-level accuracy, false alarms, and whether the perception model remains valid under the gait-induced motion and water conditions of the deployed robot.

**Figure 5:**
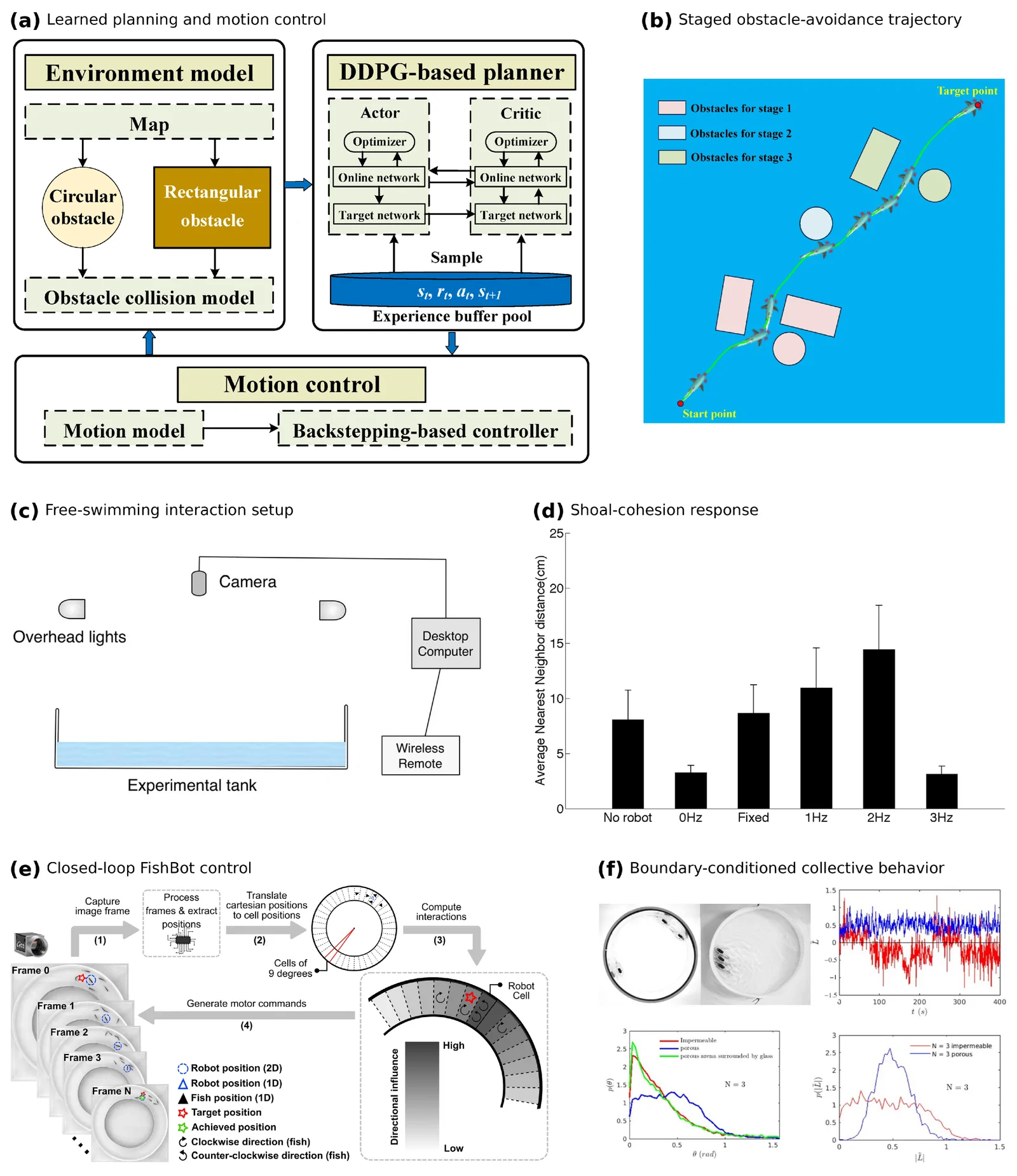
Representative examples linking autonomous planning, closed-loop interaction, and collective task outcomes. (**a**) Environment model, DDPG-based planner, and motion-control architecture, adapted from Wang et al. [115], Figure 3, under CC BY 4.0. (**b**) Staged obstacle-avoidance trajectory from the same study, Figure 8, CC BY 4.0. (**c**,**d**) Free-swimming robot–fish experimental setup and shoal-cohesion response, adapted from Butail et al. [108], Figures 2 and 4, under the Creative Commons Attribution license stated by PLOS. (**e**) Closed-loop FishBot control cycle, adapted from Papaspyros et al. [109], Figure 3, under the Creative Commons Attribution license stated by PLOS. (**f**) Boundary-conditioned collective organization and associated statistics, adapted from Boudet et al. [126], Figure 3, CC BY 4.0. Panels were cropped, resized, and relabeled for composition; inherited source-panel sequencing labels were removed, and negative tick labels use mathematical minus signs. Scientific content was otherwise unchanged.

Figure 5 complements the hardware-oriented evidence in Figure 4 by connecting learned planning, obstacle negotiation, controlled animal–robot interaction, and collective outcomes. The combination also illustrates an important evidence boundary: a controller architecture, an executed trajectory, an experimental apparatus, and a group-level response answer different questions and should not be treated as interchangeable demonstrations of autonomy.

In Figure 5, panels (a,b) represent the algorithm-to-trajectory chain, whereas panels (c–f) represent progressively richer interaction experiments and collective outcomes. The figure therefore complements Table 5: the table compares functional evidence, while the visual panels make the associated infrastructure, experimental boundary, and outcome level explicit.

### 4.4. Rhythmic Gait Generation and Central Pattern Generators

Central pattern generators (CPGs) provide a compact representation of rhythmic locomotion. Coupled oscillators can generate phase-locked joint or fin commands, interpolate frequency and amplitude smoothly, and support transitions among gaits [127]. In robotic fish, CPG architectures are attractive because phase relations are central to body-wave propulsion and because low-dimensional parameters can replace independent joint trajectories [128].

A CPG is a gait generator rather than a complete controller. Open-loop CPG output can produce robust periodic motion if morphology and loading are predictable, but it does not compensate automatically for current, payload, actuator degradation, or collision. Feedback can enter through oscillator frequency, amplitude, phase, bias, or coupling. The design question is which variable should be adapted and which sensor makes that variable observable.

Multi-task and mode-switching CPG systems demonstrate that one oscillator network can support transit, turning, diving, or different body-wave patterns [129,130]. Their evaluation should include transition time, overshoot, phase continuity, repeatability, and the effect of body dynamics. A commanded smooth transition is not sufficient if the compliant body temporarily loses stability or if the actuator saturates. CPG parameters should also be interpreted together with measured kinematics rather than treated as direct biological variables.

Optimization can tune gait parameters, but a best value found in one tank is not necessarily transferable. Onboard and data-driven gait optimization have shown that physical experiments can be used to improve speed or other objectives [131]. To support a design claim, the search boundary, number of evaluations, environmental conditions, and sensitivity around the optimum should be reported. A broad robust region is usually more useful than a narrow maximum.

### 4.5. Model-Based Estimation and Control

Model-based control uses an explicit representation of robot and environment dynamics. Models range from kinematic mappings between joint phase and body wave to reduced-order rigid-body models, identified input–output models, and fluid–structure simulations. Their appropriate complexity depends on the purpose. A detailed model may explain a mechanism, while a reduced model may be better suited for onboard state estimation or predictive control.

Data-driven dynamic models can capture the effective relationship between gait parameters and robot motion when first-principles hydrodynamics are too expensive or uncertain [132]. Such models remain local to the morphology, payload, and training conditions. Validation should therefore include held-out gaits and disturbances, not only reconstruction of the training set. When a model is used for control, prediction accuracy should be connected to tracking, stability, or energy outcomes. The mechanism chain introduced in Section 2 can be interpreted as a control-oriented dependency mapping from structural parameters and forcing to realized deformation and then to force, motion, and energy outputs. A complete state-space model, however, is platform-specific because fluid–structure and sensing feedback introduce closed loops; representing the cross-platform framework as a universal directed acyclic graph would therefore imply a stronger and often incorrect causal structure.

Classical feedback remains important. Heading, depth, speed, and trajectory can be regulated with PID or related controllers when the relevant state is observable and the operating envelope is moderate. More advanced adaptive or predictive methods are justified when hydrodynamic parameters change, actuator limits matter, or multiple objectives must be coordinated. Algorithmic sophistication should not replace a clear baseline. A simpler controller with reliable onboard sensing may outperform a more complex controller that depends on external motion capture or a narrow model.

The physical body can reduce or increase control burden. Passive fin feathering, distributed compliance, or inertial stability may reject disturbances mechanically. Conversely, soft-body hysteresis and unmodeled phase lag can destabilize feedback. A useful model-based study separates the performance gain due to control from that due to morphology, for example through fixed-body or open-loop baselines.

Trajectory-tracking studies have combined CPG gait generation with model predictive control [114], while related tail-design work connects the mechanical transmission to the same tracking objective [133]. A closed-loop sensory-feedback CPG provides a complementary architecture in which sensory regulation modifies the rhythmic generator directly [113].

### 4.6. Learning-Enabled Control and Design

Learning methods are relevant here when they address the embodied control, dynamics, perception, or design of a fish-like robot. Hydrodynamics, compliant-body mechanics, and sensor signals are strongly nonlinear, but the central evidence question is whether an algorithmic gain survives physical transfer. Claims of learning-enabled advantage are therefore most interpretable when training environments, observations, actions, randomization ranges, reset procedures, safety constraints, external tracking, and conventional or model-based baselines are reported explicitly.

Simulation-to-real transfer is particularly sensitive to stiffness, damping, fin deformation, actuator delay, payload, and sensor drift. Learning may also optimize morphology, stiffness, actuator placement, or gait, but a numerically optimized design remains a hypothesis until fabricated and tested. Accordingly, the evidentiary value of learning-enabled results depends more on physical deployment, matched baselines, disturbance coverage, and disclosure of sensing/computation infrastructure than on model complexity alone.

#### Recent Fish-Specific Learning Evidence

Recent fish-specific learning studies increasingly connect learned dynamics and control to physical robotic platforms. Rodwell and Tallapragada used a physics-informed curriculum for simulated velocity/path tracking, providing a data-efficiency example without physical-robot transfer [134]. Wang et al. validated learning-based discontinuous path following on a biomimetic vehicle with undulatory fins, Chen et al. trained a lightweight RL–CPG heading controller directly on an embedded robotic fish and tested flow and damaged-tail cases, and Zhong et al. learned an ANN dynamics model from robotic fish trajectories before transferring a deep-RL controller to the physical robot [116,117,135]. The broader 2026 review of reinforcement-learning-enabled robotic fish contextualizes this trend [123]. Across these studies, transferability remains conditioned by localization, training resets, disturbance distributions, compute/power cost, and comparison with tuned conventional control.

Adjacent AI-enabled fluid diagnostics can also clarify mechanisms or evidence boundaries. For example, the PINN reconstruction of pressure around swimming fish by Calicchia et al. [136] informs hydrodynamic inference and potential lateral-line modeling, but it represents offline inference rather than demonstrated onboard autonomy. Cross-domain AI methods are most informative when a clear connection to fish-like robot dynamics, perception, or control can be established.

### 4.7. Biological Interaction and Multi-Robot Coordination

Robot–fish interaction creates a feedback loop in which the biological agent is part of the environment. Fish response can depend on robot size, shape, color, tail-beat frequency, trajectory, hydrodynamic disturbance, and social context. Experiments have shown that biomimetic locomotion can affect attraction and following, but the effect is species- and protocol-dependent [137,138]. Larger-school experiments further indicate that different robot attributes may dominate at different group scales [139].

Interaction studies should distinguish between attraction, absence of avoidance, following, and longer-term habituation. One positive approach response is not evidence of welfare-compatible operation. Appropriate controls include a stationary or non-biomimetic object, alternative motion patterns, and repeated trials across individuals or groups. Reporting adverse or ambiguous responses is as important as reporting attraction. Recent perspective work further emphasizes robotic swimmers as controlled tools for studying locomotor, sensory, and schooling feedback rather than only as visual mimics [140]. The robot’s wake and acoustic output may be relevant even when visual appearance is similar. Low-disturbance designs should therefore measure actuation-generated acoustic or vibration signatures when biological interaction is a mission objective rather than assuming that a soft exterior or biomimetic shape is acoustically benign. The UJIFISH-I platform provides a recent task-oriented example in which thruster avoidance and reduced mechanical noise were explicit aquaculture-inspection design objectives [141]. Direct-drive or transmission-light magnetic and piezoelectric mechanisms are plausible candidates for reducing gear-related mechanical noise because they can avoid conventional geared transmissions [65,73,74]; however, low acoustic output must be demonstrated by measured sound or vibration under the intended wet operating condition rather than inferred from actuator type alone.

Multi-robot coordination extends the autonomy problem through communication and relative localization. Underwater acoustic links are intermittent and low bandwidth; optical communication is range- and turbidity-limited; and global positioning is usually unavailable. Robust collective behavior should therefore rely on local information and degrade gracefully when communication or agents fail. Useful metrics include coverage, collision rate, communication load, localization error, detection time, and mission success after a failure. Failure tolerance should be quantified rather than asserted: examples include the probability of mission completion after *k* agent or link failures, connectivity probability, recovery time, coverage loss, and a normalized graceful-degradation curve G(k)=J(N−k)/J(N). Visually coherent schooling is not equivalent to collective task performance.

Hydrodynamic interaction among robots may provide cues or energetic benefits, but these effects are sensitive to relative phase and position. Controlled formation experiments are needed before wake exploitation is treated as a mission advantage. The same principle applies to collective adaptation: group-level gains should be separated from the performance of the best individual.

### 4.8. Onboard Computation, Communication, and Autonomy Boundaries

The autonomy boundary includes computation, localization, and communication. A robot that relies on external cameras, a tethered computer, or manually selected gaits demonstrates a different capability from a fully onboard system. External infrastructure is entirely compatible with a mechanism experiment provided that its role is stated explicitly. The EC-R/EC-T codes are therefore not proxies for autonomy: EC-R denotes integrated free swimming and EC-T a controlled task, whereas onboard/offboard sensing, localization, computation, and communication are recorded separately as infrastructure boundaries. For controlled tasks, studies that depend on external localization or offboard decision computation are classified as EC-T, or as EC-R/EC-T when integrated free-swimming performance and controlled-task execution are both directly relevant. Passive external measurement used only to observe a free-swimming mechanism does not itself create a task context. External support also precludes describing the experiment as fully onboard autonomous operation. Interpretation is strengthened when reports specify which measurements and decisions are onboard, the control and sensing rates, processing latency, communication bandwidth, and behavior after link loss.

Onboard resources create trade-offs with morphology. A large processor and battery increase rigid volume, mass, and heat, while acoustic modems and cameras affect power and geometry. Distributed low-rate sensing may be more compatible with a compliant body than a few large modules. Event-driven or hierarchical control can reduce computation by keeping fast gait stabilization local and task planning at a slower rate.

Autonomy is more informatively described through demonstrated functions and infrastructure dependence than through a single ordinal label. A platform may have onboard gait control but external localization; onboard flow estimation but manual task selection; or autonomous task selection in a structured tank. Stating these dependencies allows readers to compare systems without forcing them into a single ordinal scale.

### 4.9. Safety, Verification, and Control Assurance

Autonomy introduces failure modes that are not visible in nominal locomotion tests. An erroneous state estimate can excite an unstable body mode, a learning policy can command an unsafe frequency, and a flow sensor can interpret self-generated pressure as an obstacle or current. Control assurance therefore requires explicit operating limits and fallback behavior. Saturation bounds, frequency and curvature limits, leak or pressure alarms, communication-loss behavior, and positive-buoyancy recovery are examples of safeguards that can be evaluated independently of the task controller.

Verification is difficult because the body and environment are continuous and uncertain. Nevertheless, a layered strategy is feasible. Component tests establish sensor and actuator limits; hardware-in-the-loop tests expose timing and communication faults; controlled tank tests cover a defined grid of current, payload, and gait conditions; and task trials evaluate integrated behavior. The purpose is not to prove safety under every aquatic condition, but to define a verified envelope and show what happens at its boundary.

Learning-enabled systems require additional assurance. A policy should be tested for observation dropout, out-of-range states, actuator delay, and changes in body stiffness. A safety filter or model-based supervisory layer can constrain the action, but its intervention rate and failure behavior should be reported. If a learned controller is reset manually after failure or depends on external localization, those dependencies form part of the autonomy boundary.

Biological interaction adds ethical assurance. Control objectives that maximize attraction or following may not align with animal welfare. Biological-interaction experiments should define welfare constraints within the approved experimental protocol, including appropriate limits on approach, pursuit, contact, and task-termination conditions. Safety and welfare constraints can be incorporated as hard limits, task termination conditions, or metrics reported alongside interaction success. This treatment makes responsible interaction a control-design requirement rather than a statement added after the experiment.

### 4.10. Control Evaluation, Ablation, and Failure Analysis

An informative control experiment isolates the source of improvement. Useful ablations include removing the new sensor, replacing the proposed estimator with a simpler baseline, freezing the adaptive variable, or testing the same controller on a fixed-stiffness body. These comparisons are particularly important in embodied systems because morphology and software can compensate for one another.

Disturbance tests should specify the perturbation. A current step, lateral jet, payload shift, collision, actuator degradation, sensor dropout, and communication loss probe different mechanisms. Recovery without external intervention is stronger evidence than nominal tracking in still water. Repeated disturbances also reveal whether performance depends on one calibration.

Failure reporting should include estimator divergence, unstable gait transitions, actuator saturation, control-induced resonance, false detections, and loss of observability. A learning policy may fail outside its training range; a lateral-line estimator may fail when self-motion dominates; a CPG transition may destabilize a compliant tail. These are not merely implementation details. They define the boundary of the conditional design principle.

### 4.11. Autonomy Synthesis

Three conclusions emerge within the evidence boundaries established above. First, a sensor acquires task-level value when it improves estimation or task performance relative to an explicit baseline. Second, flow and strain measurements require calibration or modeling of self-generated signals under the realized gait. Third, autonomy is best described through demonstrated functions together with localization, computation, communication, and manual-intervention boundaries. The concluding synthesis consolidates the corresponding fish-specific design implications and discriminating ablations, while Section 5 asks whether these closed loops change task outcomes.

## 5. Task-Oriented Evaluation and Application Evidence

### 5.1. Why a Single Performance Ranking Is Insufficient

Fish-like robots are often compared through maximum speed, body lengths per second (BL/s), or tail-beat frequency. These quantities are useful, but they do not establish whether a robot is energy efficient, maneuverable, robust, autonomous, safe near organisms, or capable of completing a mission. A high-frequency flow-tank physical model and a soft ecological-observation robot may both be successful for fundamentally different reasons. Ranking them along one axis would obscure the relationship between design and purpose.

**Figure 6:**
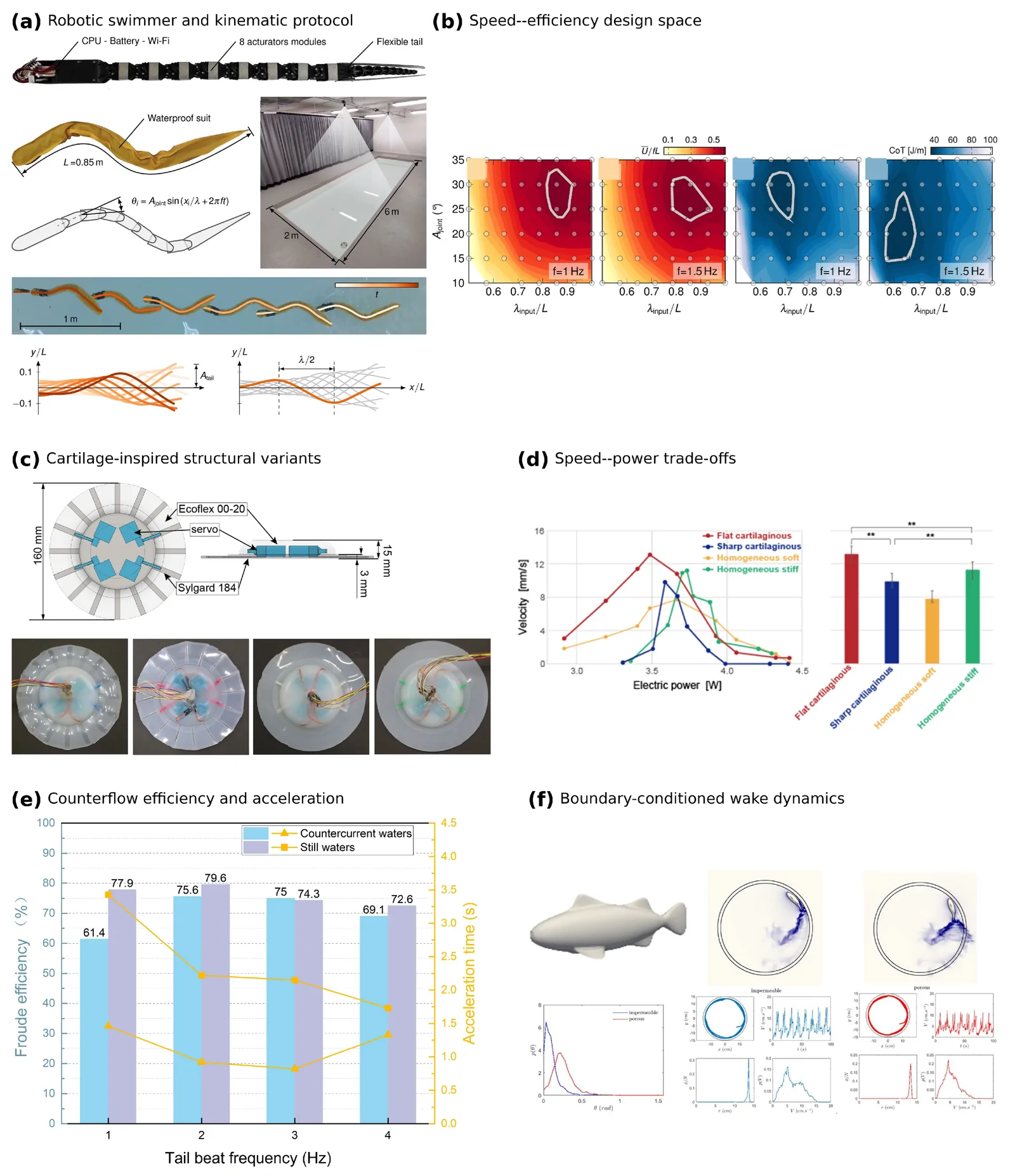
Representative examples of hydrodynamic performance, structural intervention, and protocol-sensitive validation. (**a**,**b**) Free-swimming undulatory robot, measurement protocol, and speed–cost-of-transport maps, adapted from Anastasiadis et al. [142], Figures 1 and 2, under CC BY 4.0. (**c**,**d**) Cartilage-inspired structural variants and the velocity–power comparison from the corresponding intervention, adapted from Yurugi et al. [143], Figures 4 and 8(e,f), CC BY 4.0. In panel (**d**), the double asterisks mark the statistically significant ANOVA comparisons reported by the source: flat versus sharp, $p<7.5\times 10^{-8}$; flat versus homogeneous stiff, $p<4.9\times 10^{-4}$; and sharp versus homogeneous stiff, $p<2.5\times 10^{-3}$. (**e**) Froude-efficiency and acceleration-time comparison between countercurrent and still-water cases, adapted from Gong et al. [144], Figure 5, CC BY 4.0. (**f**) Boundary-conditioned flow, wake, and motion statistics for artificial swimmers, adapted from Boudet et al. [126], Figure 6, CC BY 4.0. Panels were cropped, resized, and relabeled for composition; inherited source-panel sequencing labels and clipped label remnants were removed. Scientific content was otherwise unchanged.

Figure 6 illustrates this incompatibility using representative controlled experiments. Kinematic maps, structural interventions, electrical-power comparisons, counterflow tests, and boundary-conditioned wake measurements provide complementary evidence, but each preserves a different system boundary and causal scope.

Figure 6 is best interpreted as a map of complementary evidence boundaries, with no implication of an overall performance ranking. Panels (a,b) connect gait parameters to self-propelled speed and transport cost; panels (c,d) test a structural intervention against power and velocity; panel (e) compares operating conditions; and panel (f) shows how environmental boundaries reshape wakes and collective motion. Together, these panels illustrate why performance claims require both a controlled comparator and a clearly stated system boundary.

**Figure 7:**
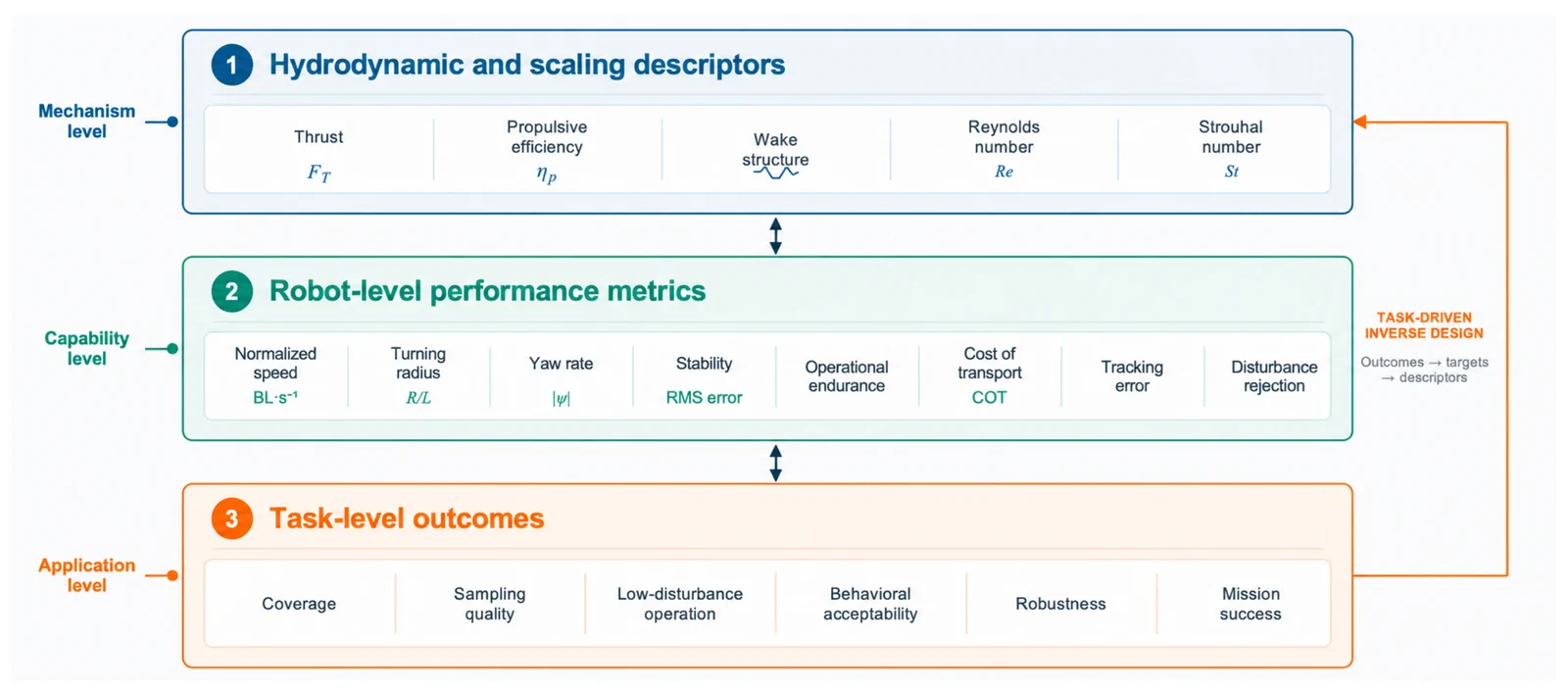
Task-oriented evaluation framework for fish-like robots. Evaluation criteria are organized from hydrodynamic and scaling descriptors at the mechanism level, through robot-level performance metrics at the capability level, to task-level outcomes at the application level. Bidirectional arrows emphasize that task outcomes should also drive the selection of required capabilities and diagnostic mechanisms.

Evaluation is more informative when explanatory descriptors, integrated capabilities, and task outcomes are separated. This distinction prevents Reynolds number, turning radius, sample integrity, and mission success from being treated as equivalent measures. Figure 7 presents a three-level bidirectional evaluation and reverse-design framework. Task priorities are specified at the mission level because their relative importance depends on the intended application.

Figure 7 translates the heterogeneous evidence in Figure 6 into a bidirectional design workflow. It separates explanatory mechanism descriptors, integrated robot capabilities, and application outcomes. Task requirements determine which capabilities matter, while capability shortfalls identify the mechanisms that should be redesigned or measured.

At the mechanism level, thrust, propulsive efficiency, wake structure, Reynolds number, and Strouhal number help explain force production, fluid regime, and scaling. At the capability level, normalized speed, turning radius, yaw rate, stability, endurance, cost of transport (COT), tracking error, and disturbance rejection describe what the assembled robot can do. At the application level, coverage, sampling quality, low-disturbance operation, behavioral acceptability, robustness, and mission success determine whether those capabilities have practical value.

The levels are connected but not interchangeable. Mechanism-level measurements help diagnose why a task succeeds or fails, while a task outcome cannot normally reveal the underlying cause on its own. Conversely, high thrust or a favorable wake does not establish mission value. The central evaluation principle is therefore conditional: a design is superior only with respect to a stated objective and evidence boundary.

### 5.2. Measurement Boundaries and Data Provenance

Speed is intuitive but protocol-sensitive. A transient maximum can exceed a sustained mean; a flow-tank value may be relative to water rather than ground; and a short optimized interval may not represent mission cruising. An interpretable report states whether the robot is free swimming or constrained, the averaging interval, body-length convention, payload, water conditions, and trial variability. BL/s supports cross-scale interpretation but does not correct for Reynolds number, architecture, or power boundary.

Energy comparison is even more sensitive. Mechanical power at a joint, electrical power at an actuator driver, and battery power for the complete robot answer different questions. COT is meaningful only when the numerator, mass, speed interval, and dimensional convention are explicit. For application claims, the boundary generally includes actuation, pumps or valves, onboard computation, sensing, communication, and payload. Mission energy may also contain long periods of station keeping or sampling that are not represented by steady straight swimming.

Endurance requires an operating definition. Battery runtime at idle, continuous swimming time, distance per charge, and task duration are not equivalent. Intermittent locomotion, burst-and-coast strategies, sensing duty cycles, and communication can materially change the result. Reliability likewise requires a denominator: the number of trials, failed trials, maintenance actions, and performance drift are more informative than a representative success.

Data provenance is essential when values are transferred into a review. A table entry should distinguish a directly reported value from one calculated from published variables or digitized from a graph. Derived BL/s requires compatible speed and body-length definitions; derived Strouhal number requires an unambiguous amplitude; derived COT requires a complete power boundary. When those conditions are absent, “not reported” is more accurate than a reconstructed value.

### 5.3. Straight Swimming, Hydrodynamic Performance, and Endurance

Straight-swimming tests remain a useful common module because they expose gait, speed, stability, and energy interactions. A core reporting set includes absolute speed and BL/s, body length, frequency, amplitude convention, measured body and fin kinematics, tethering, tank or channel geometry, flow condition, and repeated-trial uncertainty. If efficiency is discussed, force and power definitions must also be explicit.

Tunabot and Tunabot Flex illustrate the value of a coherent experimental family. The original platform connected high-frequency kinematics with self-propelled speed and wake measurements, while the flexible version evaluated body flexibility within a related architecture and added a transport-cost comparison [14,15]. The resulting inference is stronger than a cross-platform speed ranking because one design variable was altered under a comparatively consistent measurement boundary.

Thrust tests answer a different question from free-swimming tests. Tethered mean thrust can compare propulsors at prescribed motion, but free speed emerges when thrust balances body drag and when the controller, body recoil, and stability are included. Propulsive efficiency measured on a fin rig should not be equated with whole-system electrical efficiency. Both measurements are useful when their scope is preserved [29].

Endurance moves evaluation from propulsion toward the complete system. Battery energy, actuator and driver efficiency, buoyancy control, sensors, communication, computation, and mission behavior all contribute. A robot that moves slowly but collects stable measurements over a long mission may be more effective than a faster platform with limited operational duration. Reporting distance, duty cycle, and task completion alongside runtime improves interpretability.

### 5.4. Maneuverability, Stability, and Disturbance Response

Maneuverability includes turning, braking, reversing, hovering, depth change, obstacle negotiation, and recovery after interaction. Minimum turning radius alone is inadequate because it can be achieved at very low speed or with large post-turn oscillations. A stronger report combines normalized radius R/L, yaw rate, entry speed, trajectory error, depth and roll excursion, settling time, and repeated trials.

MPF mechanisms and compliant fins are especially relevant at low speed because they can redirect force without large body curvature. Passive feathering can reduce active degrees of freedom, but bench deformation does not establish whole-robot maneuverability [20]. The final evidence must connect fin motion to robot trajectory and stability under a stated stroke and speed.

Stability must be defined relative to the task. Relevant quantities include heading variance during transit, depth or position error during sampling, roll and pitch during imaging, and station-keeping error in current. A high-frequency tail may preserve a stable mean heading while inducing oscillations that degrade camera or sensor quality. A compliant body may reduce high-frequency disturbance while introducing slow drift. Reporting both RMS and peak values, settling time, and measurement bandwidth helps distinguish these behaviors.

Disturbance-response experiments should state the perturbation. A current step, lateral jet, payload shift, collision, sensor dropout, and actuator degradation probe different capabilities. Recovery without external intervention is stronger evidence than nominal tracking in still water. Repeated tests also reveal whether the behavior depends on one calibration or initial condition.

### 5.5. Application Domains and Their Evaluation Priorities

#### 5.5.1. Ecological Observation and Biological Interaction

Ecological observation prioritizes low disturbance, safe proximity, stable imaging, and mission duration. SoFi demonstrated the relevance of matching a soft architecture, acoustic command system, and onboard camera to marine observation rather than optimizing solely for speed [62]. A complete evaluation would include approach distance, observation duration, image stability, wake or acoustic disturbance, control reliability, leakage or maintenance, and organism response.

Robot–fish interaction adds behavioral outcomes. Attraction, following, avoidance, and habituation are distinct phenomena and can depend on locomotion, size, color, trajectory, and social context. Biomimetic tail motion can affect response in some experiments, while larger-group studies indicate that robot size and speed may dominate in other settings [137,139]. Conclusions should therefore remain species- and protocol-specific. Appropriate controls, repeated trials, welfare procedures, and adverse responses are required before “compatibility” is claimed.

Low disturbance cannot be inferred from appearance or a soft exterior. Hydrodynamic wake, acoustic emissions, illumination, contact force, and repeated exposure may all matter. When acoustic disturbance is relevant, reports should characterize background and active-gait sound or vibration over the frequencies resolved by the instrumentation, with propulsion state and measurement geometry stated. Recent aquaculture-oriented robotic fish work explicitly treats reduced mechanical noise and thruster avoidance as task-level design requirements [141]. For ecological systems, task success may therefore combine usable observation time with a biological-response constraint rather than using locomotion performance alone.

#### 5.5.2. Monitoring, Inspection, and Environmental Sampling

Water-quality monitoring requires spatial coverage, localization, sensor calibration, endurance, and a measurement boundary that accounts for the robot’s wake. Sensor placement relative to the tail and body can affect whether the robot measures ambient water or a self-disturbed region. Useful task outcomes include mapped area, measurement uncertainty, data completeness, and energy per surveyed area.

Infrastructure and aquaculture inspection add boundary following, obstacle clearance, image stability, collision tolerance, and anomaly detection. Artificial lateral-line sensing has supported wall-relative control and near-field detection in controlled settings [48,106]. Recent underwater visual-inspection studies show that the downstream perception task also depends on image enhancement, defect detection or segmentation, viewing geometry, and motion/visibility conditions [124,125]. UJIFISH-I further illustrates the integration of biomimetic propulsion, inspection, teleoperation, and sensor deployment in aquaculture [141]. These adjacent inspection results strengthen the task definition but do not by themselves prove a fish-like morphology advantage. Field transfer requires irregular geometry, variable currents, turbidity, fouling, and failures to be included. A wall-following trajectory in a regular tank is task-relevant evidence, but not yet a complete aquaculture inspection mission.

Sampling adds physical interaction and payload change. Pumps, bottles, manipulators, or sediment collectors alter buoyancy, drag, center of mass, and station-keeping demand. Arrival at a sampling point is not the final outcome. Sampling success rate, sample integrity, contamination control, positioning error during collection, payload effect, and reliable recovery are more direct measures of application value.

#### 5.5.3. Transit, Gait Adaptation, and Multi-Purpose Missions

High-performance swimming platforms are valuable for identifying the limits of oscillatory propulsion and for testing flexibility–frequency relationships. Recent robotic tuna and miniature swimmers broaden the scale and implementation space [105,145]. Their speed claims are most useful when accompanied by length, frequency, payload, stability, and energy context. They should not be interpreted as a universal ranking of all robotic fish.

Multi-purpose missions move among transit, approach, maneuvering, and station keeping. A reconfigurable or variable-stiffness platform can potentially select different deformation distributions for these phases [16,76]. The application metric is not simply the best gait. Transition time, energy, repeatability, stability during reconfiguration, sensing requirement, and autonomous selection determine whether adaptability has been demonstrated. Manual reconfiguration establishes mechanism potential; closed-loop selection under task conditions establishes adaptive capability.

#### 5.5.4. Swarm and Collective Tasks

Multiple robotic fish can increase coverage, redundancy, and resilience, but underwater communication and localization remain restrictive. A visually coherent school does not by itself demonstrate collective utility. Evaluation should include coverage efficiency, detection or completion time, collision rate, communication load, localization error, and performance after communication or agent failure. Quantitative failure-tolerance reporting can include mission-success probability after *k* node/link failures, connectivity probability, recovery time, coverage loss, and the normalized graceful-degradation function G(k)=J(N−k)/J(N). As an external methodological analogue rather than direct evidence from fish-like robots, the lifetime-based probabilistic functional-dependence framework of Da et al. [146] provides a transferable way to reason about interacting communication-node failures over time; application to robotic fish swarms requires revalidation of sensor–relay–sink topology, mobility, functional-dependence, communication, and recovery assumptions before system-level reliability is inferred.

Hydrodynamic interactions can act as disturbances, cues, or potential energetic opportunities. Any claimed energy benefit requires controlled relative positions and phases, together with a comparison against independent swimming. Group-level gains should also be separated from the performance of the best individual. A robust swarm is one that degrades gracefully when links are intermittent or a member fails.

A recurring field-validation gap remains: controlled task demonstrations and biological-interaction experiments are available, whereas repeated long-duration missions with standardized reliability reporting remain uncommon.

Application-oriented prototypes illustrate why task outcomes cannot be inferred from locomotion alone. A 3D-printed robotic fish integrates water-quality sensors and autonomous cruising functions [107]. A water-entry strategy addresses recovery from stranding rather than steady swimming [147]. High-speed leaping research evaluates transition and exit performance outside the usual tank-cruising benchmark [148]. Wireless gliding fish trade transit speed for compact actuation and controllable buoyancy-assisted motion [149].

### 5.6. Reverse-Design Cases Linking Task Outcomes to Embodiment

#### 5.6.1. Ecological Observation

The primary outcomes are usable image time, minimum approach distance, low acoustic and hydrodynamic disturbance, absence of adverse biological response, and mission duration. These outcomes imply stable low-speed posture, smooth approach, collision-safe surfaces, quiet actuation, and an imaging payload that does not destabilize the body. A plausible embodiment is a rigid–soft hybrid with compliant propulsion and independent depth or trim control, rather than the globally softest body. Validation should report image motion, acoustic output, wake or flow disturbance, animal response, leakage, battery endurance, and repeated approaches; SoFi- and UJIFISH-like demonstrations establish feasibility and task-oriented integration but not a universal ecological advantage [62,137,138,139,141].

#### 5.6.2. Near-Wall Structure Inspection

The task outcomes are coverage, defect-detection quality, wall-distance error, collision rate, and completion under current. These require controllable transit, low-speed lateral authority, stable illumination, localization, and flow-relative feedback. A BCF propulsor can provide transit while paired fins or differential body control provide local maneuvering; a pressure array may assist near-wall estimation after self-motion compensation. The discriminating experiment compares the same payload and route with and without flow sensing across wall geometries and currents, reporting missed regions and failures rather than only nominal tracking [47,48,112].

#### 5.6.3. High-Speed Transit

The task is to cover a specified distance within an energy or time budget, not to achieve one transient maximum. Required capabilities include sustained normalized speed, heading stability, acceptable whole-system COT, and fatigue resistance. This favors a tail-dominant body with low-backlash transmission and a posterior compliance distribution matched to the operating frequency. Validation requires a stiffness–frequency sweep, measured centerline phase, continuous rather than peak speed, complete power accounting, and post-test structural inspection [14,15,38,58].

These cases illustrate reverse design: task outcomes define capability requirements, which constrain mechanisms and embodiment. They also expose missing evidence. A robot that meets a capability metric in an open tank may still fail when payload, boundary proximity, current, or biological response is introduced.

### 5.7. Using the Framework for Design Decisions

The evaluation framework is intended to support prospective design decisions as well as retrospective comparison. A project can begin with task-level outcomes, identify the robot capabilities required to achieve them, and then select the mechanism-level descriptors needed for diagnosis. For ecological observation, low-disturbance operation and stable imagery may translate into requirements on attitude oscillation, approach speed, wake, and acoustic output. For environmental sampling, sample integrity and task success may instead emphasize station keeping, payload compensation, and endurance. This reverse-design route yields a narrower, more task-focused metric set and avoids indiscriminate reporting of every quantity available in the laboratory.

The same logic can reveal when a proposed innovation is unlikely to matter. An actuator improvement that increases no-load frequency may have little mission value if the task is limited by station keeping or sensing. A new flow estimator may be unnecessary if vision remains reliable and pressure ports create substantial maintenance. Conversely, a modest speed reduction may be acceptable if a compliant body improves camera stability, collision tolerance, or biological response. Such decisions require explicit priorities rather than a generic assumption that more speed, softness, or autonomy is always better.

Cross-platform comparison remains useful when it is framed as a design-space map rather than an overall ranking. Grouping platforms by task, evidence context, scale, and system boundary makes common trends and missing evidence visible without forcing incompatible outcomes onto one axis. A high-performance physical model can isolate a stiffness–frequency mechanism, whereas a field robot can reveal integration and maintenance constraints. The framework preserves both forms of evidence while keeping their inference scopes distinct.

### 5.8. Failure Modes and Field Readiness

Field readiness is a property of the integrated system. It includes propulsion, buoyancy, sealing, sensing, computation, communication, task payload, recovery, and maintenance. A platform may provide direct evidence for one function while leaving others less validated. Evidence-context reporting allows the contribution to be recognized without requiring every study to perform an open-water deployment.

Mechanical failures include tendon stretch or rupture, fin tearing, skin delamination, seal leakage, joint backlash, corrosion, and actuator overheating. Sensing failures include bias drift, fouling, saturation, damage, and loss of calibration under self-motion. Control failures include estimator divergence, unstable gait transitions, saturation, and dependence on external localization. Mission failures include missed samples, collisions, communication loss, unrecoverable groundings, or inability to return. Field-oriented experimental reports should include failure categories and recovery behavior rather than only successful trials.

Environmental robustness can be evaluated progressively through current speed and direction, turbulence, turbidity, lighting, boundary geometry, temperature, salinity, payload, and duration. Field interpretation is strengthened by reporting local ambient-flow magnitude, direction, and temporal variability using a calibrated method appropriate to the scale; ADV or ADCP measurements are appropriate where their sampling volume and deployment geometry fit the experiment, but they are not the only admissible methods. Longer tests reveal battery and thermal behavior, material creep, fatigue, and calibration drift. Recovery measures, such as positive buoyancy after power loss or an emergency acoustic command, belong to mission design when field readiness is discussed.

### 5.9. Recommended Benchmarking and Reporting Elements

The evidence synthesized above suggests a common reporting core supplemented by task-specific modules. The common core includes:

- Body length convention, mass, buoyancy, joint count, fin geometry, material, and stiffness characterization;
- Actuator, transmission, power source, onboard electronics, payload, tethering, and energy boundary, including synchronized bus-voltage/current measurement with calibrated DAQ hardware (or an equivalently calibrated acquisition system) when whole-system electrical power is claimed;
- Tank, channel, or field geometry; water depth; ambient-flow magnitude/direction and measurement method; temperature and salinity where a hydrodynamic comparison is intended; and test duration;
- Commanded and realized frequency, amplitude, phase, curvature, speed, and repeated-trial uncertainty;
- Sensor placement, calibration, localization source, control rate, processing method, software dependencies, and explicit onboard/offboard computation and communication boundaries;
- Failure events, excluded trials, leakage, fatigue, maintenance, and recovery strategy.

Task modules then add appropriate outcomes. A transit module includes COT and stability; a maneuvering module includes R/L, the yaw rate, braking, and settling; a sensing module includes accuracy, range, false alarms, and self-motion effects; a sampling module includes success and sample integrity; an interaction module includes behavioral response and welfare controls; and a swarm module includes collective success and failure tolerance.

The two main intervention evidence tables use R, D, G, NR, and IC only as table-level provenance labels so that reported values, transparent calculations, graph-digitized values, missing fields, and incompatible conditions are not conflated. Not every paper must report every metric, but every comparative claim requires the context needed to interpret it. Standardization is not intended to force all robots into one objective; it creates a common measurement boundary within which task-specific designs can be compared fairly.

### 5.10. Metric Definitions and Comparison Boundaries

The layered framework is complemented by explicit definitions in the surrounding text. Each quantity must be interpreted together with its length convention, amplitude definition, power boundary, localization source, and test environment.

Mechanism-level descriptors are mainly explanatory. The Reynolds number indicates a flow regime, and the Strouhal number summarizes a frequency–amplitude–speed relationship; neither determines application suitability. Wake structure can explain momentum transfer or disturbance, but its interpretation depends on three-dimensional flow, body drag, and power. These descriptors are most useful when they explain why a controlled change affects a robot-level capability.

Capability metrics should generally be treated as a vector. A robot can be fast but unstable, efficient at one speed but inefficient over a mission, or agile but difficult to localize. Pareto comparisons are often more informative than a weighted scalar because task priorities differ. If a composite score is used, the normalization, weights, and sensitivity to those choices must be disclosed.

Task outcomes require operational definitions. “Coverage” may mean area visited, area observed at sufficient sensor quality, or probability of detecting a target. “Mission success” may refer to one demonstration or a success rate over repeated attempts. “Behavioral acceptability” may denote attraction, the absence of avoidance, or an explicitly welfare-compatible response. The outcome definition must precede the experiment.

### 5.11. Evaluation Synthesis

Mechanism descriptors explain why a system behaves as it does, robot-level metrics establish capability, and task outcomes determine application value. Figure 7 is bidirectional because mission requirements should also determine which capabilities and mechanisms deserve measurement or redesign. Cross-platform normalized values remain descriptive unless definitions and protocols are compatible, while controlled interventions provide the strongest basis for causal design claims. Graph-digitized values are retained only when the source figure provides interpretable axes, units, and quantity definitions; uncalibrated video displacement, unspecified playback rate, battery-capacity division, and motor nameplate power remain NR rather than being converted into artificial precision. These evaluation rules are carried into the concluding synthesis together with the reliability and autonomy boundaries developed above.

## 6. Consolidated Design Principles, Contradictions, and Research Agenda

### 6.1. Evidence-Informed Conditional Principles

A transferable design principle must specify both the conditions that support it and the conditions under which it may fail. Table 6 therefore consolidates recurring claims from Section 2, Section 3, Section 4 and Section 5 into a single evidence matrix. For each principle, the table separates supporting from limiting evidence, states the applicability conditions, records the relevant evidence context, and proposes a discriminating validation test. No formal confidence score is assigned; evidentiary strength is conveyed through the intervention type, comparator, operating boundary, and unresolved limitations.

Several conclusions have comparatively direct support. Wet compliance modifies realized underwater kinematics and can improve performance within a defined operating range; commanded motion cannot substitute for measured deformation; and flow sensing acquires task-level value when it is linked to an observable variable and evaluated in closed loop. Other claims remain less mature. Online variable stiffness expands the attainable operating space, but whole-system adjustment energy, transition cost, and reliability are rarely measured. Likewise, task-oriented architecture selection lacks sufficient matched same-mission comparisons with alternative robotic fish architectures or conventional underwater vehicles. These claims are therefore retained as conditional hypotheses for design rather than treated as settled advantages.

### 6.2. Contradictions and Unresolved Quantities

Much of the apparent disagreement among stiffness studies reflects inconsistent definitions and experimental boundaries rather than direct contradiction. Studies probe different body regions, wet natural frequencies, actuation modes, payloads, and stiffness measures. A soft material can form a stiff structure through geometry; a nominally rigid body can contain a compliant peduncle; and a variable-stiffness mechanism can add enough mass or damping to offset its theoretical benefit. Stiffness-centered claims are most interpretable when *E*, geometry, EI(x) or equivalent joint stiffness, wet natural frequency, damping, phase, and realized curvature are reported together.

Efficiency evidence is fragmented for the same reason. Tethered thrust, self-propelled speed, quasi-propulsive efficiency, actuator efficiency, electrical $COT_{g}$, $E_{d}$, drag-normalized $COT_{D}$, and whole-system endurance describe different boundaries. A favorable wake or thrust increase can coexist with poorer mission energy after drivers, pumps, computation, and payload are included. The most informative next step is therefore to measure mechanism, robot capability, and task outcome on the same platform whenever possible instead of inferring one level from another.

Autonomy evidence remains concentrated in structured laboratory tasks. Pressure feedback can support wall following or flow-relative control, and recent studies of fish-like robots extend learning from policy optimization to learned dynamics and physical deployment [117,123]. Transfer across gaits, geometries, currents, sensor drift, training distributions, and infrastructure remains the larger uncertainty. External localization or offboard computation does not invalidate a result, but it changes the capability being demonstrated. Reporting these dependencies alongside the EC context allows task-level evidence to be interpreted without conflating it with fully onboard autonomy.

### 6.3. Priority Experiments and Validation Needs

Three experiment classes would resolve several of the most consequential gaps. First, a factorial same-platform stiffness–frequency–payload study should measure wet dynamics, centerline curvature, phase, thrust or a towing reference, whole-system power, speed, $COT_{g}$, $E_{d}$, and, where a matched towing reference is available, $COT_{D}$. Second, an onboard sensing ablation should compare no sensing, sensing without self-motion compensation, and sensing with compensation across multiple currents and boundary geometries. Third, reverse-designed mission trials should compare at least two architectures—or a robotic fish and a conventional platform—under the same payload, route, environment, duration, and success criterion.

These experiments target gaps that cannot be closed by additional peak-performance demonstrations alone. BCF propulsion, tail compliance, and bounded laboratory control are supported by a broader set of direct experimental studies than long-duration deployment, maintenance, biofouling, welfare, or autonomy under changing field conditions. The latter topics should therefore be treated as priority validation problems whose design rules remain provisional.

### 6.4. Limitations

This critical review inherits several limitations from the available evidence. Quantitative protocols remain heterogeneous, restricting direct cross-platform comparison and precluding pooled effect estimates. Matched same-platform interventions are still limited for important claims concerning whole-system energy benefit, task-level architecture comparison, and long-duration reliability. The reporting of uncertainty, failures, maintenance, payload, and power boundaries is inconsistent. Field evidence is also uneven: BCF propulsion, tail compliance, and laboratory control are better represented than MPF integration, buoyancy and depth-control systems, acoustic navigation, long-duration ocean deployment, biofouling, maintenance, and animal-welfare endpoints. Non-fish aquatic analogues are included only when both the transferable mechanism and its applicability boundary can be stated explicitly.

The article is a critical synthesis rather than a systematic review or meta-analysis. Its purpose is to identify transferable mechanisms, evidence boundaries, and unresolved design questions, not to claim exhaustive bibliometric coverage or pooled quantitative effects. The resulting principles should therefore be read as evidence-bounded design propositions whose validity must be tested within stated operating and mission conditions.

### 6.5. Conclusions

The five questions posed in Section 1.2 converge on one conceptual result: the transferable unit of fish-like biomimetic design is a functional mechanism, not external resemblance. A mechanism becomes engineering knowledge when it can be expressed through measurable descriptors, reproduced in a robotic embodiment, and observed in the realized in-water configuration. Morphology, actuation, compliance, sensing, control, power, and payload determine whether that mechanism survives integration, completing the mechanism-to-evidence progression summarized in Figure 1.

The reviewed evidence also shows why mechanism, capability, and task outcome must remain distinct. Component, free-swimming, controlled-task, and field or biological-interaction studies support different scopes of inference; local gains in strain, thrust, tracking, or peak speed do not automatically translate into mission value. Task requirements should instead determine the capabilities that matter and, in turn, the mechanisms that require measurement or redesign. This logic is expressed in the bidirectional framework of Figure 7 and in the nine conditional principles of Table 6.

The practical implication is a shift in how fish-like robots are compared and developed. Stronger design claims require controlled interventions, measurements of realized underwater kinematics, explicit definitions of whole-system power and autonomy boundaries, matched comparisons at the task level, and validation under intended mission conditions. Biomimetic transfer is therefore most valuable when a functional mechanism retains a measurable advantage after full robotic integration and task-level validation. 

## Figures and Tables

**Table 1:** Qualitative positioning of the present review relative to representative prior reviews. The comparison identifies major thematic emphases and is not intended as an exhaustive or quantitatively coded bibliometric assessment. A check mark denotes substantial dedicated coverage; “partial” denotes subsection-level or passing treatment; “–” denotes no explicit treatment.

Review	Primary Organizing Logic	BCF/MPF	Soft/Variable Stiffness	Sensing/Closed Loop	Task Outcomes	Evidence Contexts	Main-Text Evidence Linkage	Distinctive Contribution/Remaining Gap
Raj and Thakur (2016) [1]	Design, sensing, actuation, autonomy	√	partial	√	partial	–	–	Broad multidisciplinary entry point; task-conditioned evidence tracing is not its primary organizing emphasis.
Scaradozzi et al. (2017) [5]	BCF locomotion and control	√	partial	partial	–	–	–	Strong BCF emphasis; MPF, applications, and evidence-boundary analysis receive less systematic treatment.
Salazar et al. (2018) [3]	Aquatic biological/bioinspired taxonomy	√	partial	partial	–	–	–	Broad aquatic taxonomy; not a fish-like robot design synthesis.
Li et al. (2022) [4]	Comprehensive platform taxonomy	√	√	√	partial	–	partial	Extensive coverage; principle–counterevidence–condition tracing uses a different organizing logic.
Jian et al. (2022) [6]	Locomotion, control, implementation	√	partial	√	partial	–	–	Integrates implementation and control; task-level evidence structure is not a primary organizing layer.
Youssef et al. (2022) [8]	Bioinspired underwater soft robotics	partial	√	partial	partial	–	–	Strong soft-robot scope, broader than fish-like robots.
Li et al. (2024) [2]	Status and technical challenges	√	√	√	√	partial	partial	Strong engineering overview; explicit intervention-level evidence tracing is outside its main organizing scope.
Yan et al. (2025) [7]	Design, sensing, and autonomy	√	√	√	partial	partial	partial	Closest broad overlap; claims are not organized around counterevidence, task-conditioned applicability, and discriminating tests.
**This review**	**Mechanism–descriptor–evidence–task chain**	√	√	√	√	√	√	Conditional principles, counterevidence, applicability limits, discriminating tests, and main-text evidence tables with explicit source references.

**Table 2:** Evidence-context taxonomy and inference boundaries. The codes describe the scope of inference supported by an experiment; they are not a study-quality or autonomy score. For controlled tasks, dependence on external localization or offboard decision computation is classified as EC-T, or EC-R/EC-T when integrated free-swimming evidence is simultaneously relevant; passive external measurement alone does not convert a mechanism-only free-swimming test into EC-T.

Code	Evidence Context	Typical Variable-Isolation Structure	Claim Scope Best Supported	Main Limitation	Recommended Validation/Reporting Features
EC-M	Theory, computation, or reduced physical model	Defined variables under explicit model assumptions	Mechanism hypothesis, scaling trend, feasibility, sensitivity	Embodiment, unmodeled losses, and deployment not demonstrated	Validation against measurements; parameter coverage; uncertainty and convergence analysis
EC-C	Material, actuator, fin, or tethered component intervention	Controlled variable isolation within a component or tethered rig	Force, deformation, bandwidth, stiffness, sensor response, or local hydrodynamics	Cannot by itself establish free-swimming or mission benefit	Matched comparator; wet-state measurement; repetitions; uncertainty; same hardware and protocol
EC-R	Integrated free-swimming robot in laboratory water	Integrated-system coupling with multiple interacting variables	Locomotion, maneuvering, energy, or closed-loop capability of the assembled robot	Tank boundaries, payload, external infrastructure, and selected gait limit transfer	Repeated trials; dispersion; operational payload; power boundary; independent kinematic measurement
EC-T	Controlled task experiment	Task-level coupling with environmental and infrastructure dependence	Wall following, inspection, sampling, interaction, recovery, or coordinated behavior	Task geometry and support infrastructure may be narrow	Explicit baseline; task success definition; disturbances; failure reporting; ablation
EC-F	Field or biological-interaction study	Context-rich operation with limited isolation of individual mechanisms	Context-specific feasibility, biological response, maintenance, and operational integration	Mechanism isolation and generalization are difficult	Duration; environmental range; ethics; failures; maintenance; replication across sites or teams

**Table 3:** Nomenclature used for dynamic matching and energetic comparison. A subscript, “sys,” denotes the stated whole-system boundary. Characteristic scales must be reported with each derived nondimensional value.

Symbol	Definition	Typical Unit	Required Convention
*L*	Robot body length used for normalized speed	m	State whether the caudal fin is included.
$L_{c}$	Characteristic compliant length for fluid–structure matching	m	Use fin chord/compliant span for a fin-local analysis or active flexible-body length for a body-wave analysis.
*b*	Loaded span or out-of-plane breadth associated with $L_{c}$	m	Report the represented geometry.
EI(x)	Spatial bending-rigidity distribution	N m^2^	Prefer wet or in situ effective values when assembly and fluid loading alter dry values.
$f_{n,w}$	Wet natural frequency	Hz	Measure with payload, skin, wiring, seals, and intended immersion.
$r_{f}=f/f_{n,w}$	Actuation-to-wet-natural-frequency ratio	–	State the mode and operating point.
$\zeta_{w}$	Wet damping ratio	–	Identify the mode and estimation procedure.
Δϕ	Phase lag between command and realized posterior motion	rad or deg	State the command and measured landmark.
$G_{A}=A_{tip}/A_{cmd}$	Tail-tip amplitude gain	–	Use one-sided or peak-to-peak amplitude consistently.
$U_{r}$	Relative flow speed used in loading estimates	m s^−1^	Use free-stream speed for translation or a stated local/oscillatory scale for hovering and maneuvers.
$Ca_{EI}$	Hydrodynamic-to-bending Cauchy-type ratio	–	Defined here as $\rho U_{r}^{2}bL_{c}^{3}/\left(2EI\right)$; report all scales.
$P_{sys}$	Average input power over the declared system boundary	W	List actuator, driver, pump, computation, sensing, and communication inclusions.
$COT_{g}$	Gravity-normalized transport cost, $P_{sys}/\left(mgU\right)$	–	A convention, not useful-force efficiency for neutral buoyancy.
$E_{d}$	Energy per traveled distance, $P_{sys}/U$	J m^−1^	State steady or mission-averaged speed and power boundary.
$D_{tow}\left(U\right)$, $COT_{D}$	Matched towing resistance and $P_{sys}/\left[D_{tow}\left(U\right)U\right]$	N; –	Require the same non-propulsive configuration and speed.

**Table 4:** Representative intervention-oriented, same-platform, or closely related-platform evidence for morphology, actuation, and compliance. For values presented in this table, R denotes directly reported, D transparently derived, G graph-digitized, NR not reported or not unambiguously traceable, and IC protocol-incompatible. Evidence-context codes use the EC prefix and therefore cannot be confused with data status.

Study	Intervention/Comparator	Endpoint and Data Status	EC	Inference and Applicability Boundary
Tunabot Flex [15]	Flexible-body variant relative to a related tuna-inspired platform	Speed and COT changed with flexibility; same-platform scalar comparison IC	EC-R	Related rather than identical platforms; causal attribution requires a same-platform wet-dynamic intervention.
Deformable caudal-fin rig [50]	Controlled deformation versus undeformed command on the same tethered rig	Mean thrust +18.2% (R) and +27.5% (R), depending on deformation mode	EC-C	Supports a rig-level mean-thrust effect, not a free-swimming speed or whole-system efficiency gain.
Tunable-stiffness tuna [38]	Posterior stiffness settings on one robot	Maximum 0.70 m s^−1^ (R), 2.0 BL s^−1^ (D); energy trend with frequency (R)	EC-R	Optimum depends on morphology, frequency, wet dynamics, payload, and power boundary.
Tunable caudal peduncle [39]	Peduncle stiffness settings on the same platform	0.091 m s^−1^ (R), 0.16 BL s^−1^ (D) at the reported optimum	EC-R	Tail-actuation energy alone excludes pump/adjustment and whole-system cost.
Tendon-driven T-Fish [91]	Passive-tail settings jointly optimized on one body	0.47 m s^−1^ (R); R/L=0.31 (R)	EC-R	Tank-specific optimum; transfer requires payload and trial-dispersion information in addition to the reported performance metrics.
TenFiBot [92]	Antagonistic tensegrity stiffness modulation	Approximately 14-fold stiffness range (R); speed effect IC across settings	EC-C/EC-R	Demonstrates controllable structural range, not automatic performance improvement; mass, hysteresis, and wet repeatability matter.
Hydraulic variable-stiffness fish [93]	Online hydraulic stiffness selection	0.389 m s^−1^ (R), 0.63 BL s^−1^ (D), R/L=0.14 (R)	EC-R	Hydraulic supply, sealing, adjustment power, and maintenance belong to the system boundary.
Online hydraulic tail [40]	Within-stroke modulation versus fixed settings	Thrust/efficiency Pareto improvement (R); scalar effect NR	EC-C/EC-R	Quantitative transfer requires source curves, repeatability, and complete hydraulic power.

**Table 5:** Representative sensing and closed-loop task evidence, grouped by function. R/D/G/NR/IC denote table-level data provenance; EC-R and EC-T denote integrated-robot and controlled-task evidence contexts. EC codes are not autonomy scores; onboard/offboard sensing, localization, computation, and communication are disclosed separately in the final column.

Function/Study	Intervention	Comparator	Endpoint and Data Status	EC	Autonomy/Transfer Boundary
Flow-relative holding [112]	Pressure-derived relative-flow feedback	Conventional position behavior/higher-flow region	Station holding and reduced energy use in a low-flow wake; scalar effect NR	EC-T	Depends on wake geometry and experimental support infrastructure.
Speed regulation [47]	Multi-point pressure sensing and amplitude feedback	Open-loop gait	Prescribed-speed regulation demonstrated; closed-loop error NR	EC-R/EC-T	Self-generated flow and gait-specific calibration limit transfer.
Wall following [48]	Surface-pressure feedback to heading control	No hydrodynamic-pressure feedback	Stable offset ratio 1.0–1.33 tail spans (R)	EC-T	Straight wall and aquarium conditions; not general obstacle navigation.
State estimation [49]	Artificial lateral line added to estimator	Model without distributed pressure information	Online state reconstruction demonstrated; comparable scalar NR	EC-R	Sensor layout and calibration determine observability.
Sensory-feedback CPG [113]	Feedback-coupled CPG	Open-loop CPG	Improved locomotion regulation reported; effect size NR	EC-R	Matched trials and uncertainty are needed for a transferable effect estimate.
CPG–MPC tracking [114]	MPC around CPG-generated gait	No matched quantitative effect-size baseline available for comparison	Circular trajectory: signed position deviation [−6.9, 14.9]% BL and heading [−4.76, 4.73]^∘^ (R); straight trajectory: [−8.6, 8.6]% BL and [−3.24, 3.55]^∘^ (R)	EC-T	Signed trajectory-specific deviations retain direction and must not be interpreted as MAE, RMSE, unsigned error magnitude, or a relative improvement percentage; localization boundary must be disclosed.
Safe exploration [115]	Learned local planner plus backstepping yaw control	Non-learning/local-planning baseline	Collision-aware exploration reported; success-rate effect NR	EC-T	Controlled obstacle fields; training distribution and external infrastructure matter.
Real-world RL heading control [116]	Lightweight RL decision layer with CPG action execution	Tuned PID controller	Physical-flow and damaged-tail tests reported; transferable scalar improvement NR	EC-T	Direct physical training on embedded hardware; transferability is bounded by platform, flow range, failure severity, and reset protocol.
Learned dynamic model + DRL [117]	ANN dynamics model learned from physical trajectories; PPO policy transferred to robot	Surrogate/model-based approach discussed in source	Goal-reaching physical tests with reported positional accuracy 0.018 m (0.075 BL) and orientation tolerance 0.128 rad (R)	EC-T	Learned model and policy remain bounded by training trajectories, localization, target task, and sim-to-real mismatch; state the onboard/offboard inference boundary.
Turbulent-plume tracking [118]	Differential onboard pressure and learned gradient policy	Random search	Jet location at more than 2× the random-search rate (R)	EC-T	13,000-L tank task; peer-reviewed proof of concept, not ocean deployment.

*Note: Signed trajectory-specific deviations retain direction and must not be interpreted as MAE, RMSE, unsigned error magnitude, or relative improvement percentage.*

**Table 6:** Consolidated evidence-informed conditional design principles. The table summarizes supporting and limiting evidence, applicability conditions, evidence context, and a discriminating validation test. EC codes indicate inference context rather than a formal confidence score.

Conditional Design Principle	Supporting and Limiting Evidence	Applicability Conditions	Evidence Context	Discriminating Validation Test
Match stiffness to actuation frequency and wet loading within a defined operating range rather than maximizing flexibility.	Support: wet stiffness interventions and related-platform comparisons [15,25,38,39]. Limit: excess compliance can increase phase lag, damping, and ineffective lateral motion [35,36,94].	Geometry, wet natural frequency, payload, and gait range defined.	EC-M/C/R	Same-platform stiffness–frequency sweep measuring curvature, phase, complete power, speed, and COT.
Allocate compliance spatially rather than specifying only global softness.	Support: axial stiffness and activation timing shape traveling waves [34,92]. Limit: a large global stiffness range does not establish a beneficial distribution.	Body region, joint layout, and BCF/MPF function specified.	EC-M/C/R	Compare uniform, posterior-gradient, and task-optimized distributions at identical mass and actuation.
Measure realized underwater kinematics, not only commands.	Flexible fins and active tail flexion change force through load-dependent deformation [29,30,50]; commanded frequency and amplitude can diverge from wet curvature and phase.	Synchronized kinematic and force/power measurements.	EC-C/R	Report command, measured centerline, tail-tip gain, phase lag, repeats, and uncertainty at each operating point.
Use variable stiffness only when multi-condition benefit exceeds adjustment cost.	Tunable systems expand attainable operating points [16,38,40,93]; complete adjustment-energy and reliability accounting is sparse, while added mass, pump/high-voltage demand, hysteresis, leakage, and control complexity can erase gains. The present evidence therefore remains provisional for net whole-system mission benefit.	Mission contains materially different operating conditions.	EC-C/R	Compare fixed-best, fixed-compromise, and adaptive stiffness using synchronized whole-system power, adjustment energy, transition time, reliability, and mission success.
Treat flow sensing as a closed-loop intervention and explicitly account for self-motion contamination.	Pressure feedback supports speed regulation, wall following, and state estimation [47,48,49]; sensor placement, gait contamination, and geometry-specific calibration can dominate [41,42].	Observable state, onboard calibration, and sensor ablation reported.	EC-R/T	With/without-sensor and with/without-self-motion-model trials across gait, current, and geometry.
State autonomy and system-power boundaries explicitly.	Integrated recovery, exploration, formation, learned-control, and plume tasks expose infrastructure dependence [115,117,118,119,121]; external localization, offboard computation, and single-task fixtures narrow autonomy claims. When external localization or offboard decision computation participates in controlled task execution, the evidence context is EC-T or EC-R/EC-T rather than EC-R alone. Power interpretation likewise changes when sensing, computation, communication, or auxiliary loads fall outside the declared system boundary.	Report sensing, localization, computation, communication, energy, and intervention boundaries separately; reflect controlled-task dependence on external support in EC-T or EC-R/EC-T.	EC-R/T/F	Repeat the task without external support and under sensor/communication dropout, reporting changes in task success and power.
Judge architecture by matched task outcomes, not a single peak metric.	Observation, water-quality, aquaculture-inspection, interaction, and task studies use different endpoints [62,107,137,139,141]; matched same-mission comparisons among alternative architectures remain sparse.	Task, payload, environment, duration, and success criterion fixed.	EC-T/F	Compare alternative architectures on the same mission and environmental envelope with identical payload and success criteria.
Treat reliability as an independent response variable when readiness or mission claims are made.	Reviews identify sealing, fatigue, maintenance, and long-duration gaps [1,2,8]; short successful trials cannot estimate service life or failure probability.	Operational duration and maintenance interval specified.	EC-R/T/F	Repeated endurance cycles reporting leakage, drift, fatigue, failure, and recovery statistics.
Report complementary energy metrics rather than treating gravity-normalized COT as propulsive efficiency.	Published studies mix actuator, electrical-drive, and whole-system boundaries; the same $COT_{g}$ can conceal different auxiliary loads. A matched towing reference supports $COT_{D}$, while $E_{d}$ retains mission-level energy per distance.	Steady or mission-averaged speed, declared $P_{sys}$ boundary, and matched towing configuration when $COT_{D}$ is used.	EC-C/R/T	On one platform, report $COT_{g}$, $E_{d}$, $D_{tow}\left(U\right)$, $COT_{D}$, and endurance using the same payload and operating points.

## Data Availability

No new primary experimental data were generated in this study. Reported or derived values used in the synthesis are presented in the article with their corresponding source references.
